# Simulation and modelling of heavy metals and water quality parameters in the river

**DOI:** 10.1038/s41598-023-29878-1

**Published:** 2023-02-21

**Authors:** Maryam Khalilzadeh Poshtegal, Seyed Ahmad Mirbagheri

**Affiliations:** Faculty of Civil Environmental Engineering, K. N. Toosi University of Technology, Tehran, Iran

**Keywords:** Environmental monitoring, Pollution remediation, Environmental sciences, Hydrology, Natural hazards, Engineering

## Abstract

A one-dimensional qualitative model was created for this study’s modelling and phase transfer of the heavy metal dissolved in the river. The advection–diffusion equation considers the environmental variables temperature, dissolved oxygen, pH, and electrical conductivity as influencing parameters on the change in the concentration of the dissolved phase of heavy metals lead, cadmium, and zinc in springtime and winter. Hec-Ras hydrodynamic model and Qual2kw qualitative model have been used to determine the hydrodynamic and environmental parameters in the created model. The approach of minimizing simulation errors and Vba coding was employed to identify the constant coefficients for these relations, and the linear relation incorporating all of the parameters is thought to be the final connection. In order to simulate and calculate the concentration of the heavy metals in the dissolved phase at each site, the kinetic coefficient of the reaction corresponding to that point should be employed because the kinetic coefficient of the reaction varies in different portions of the river. Additionally, if the above mentioned environmental parameters are used in the spring and winter term of advection–diffusion equations, the accuracy of the developed model significantly improves, and the effect of other qualitative parameters is negligible, indicating that the model is effective at simulating the dissolved phase of heavy metals in the river.

## Introduction

**Figure 1:**
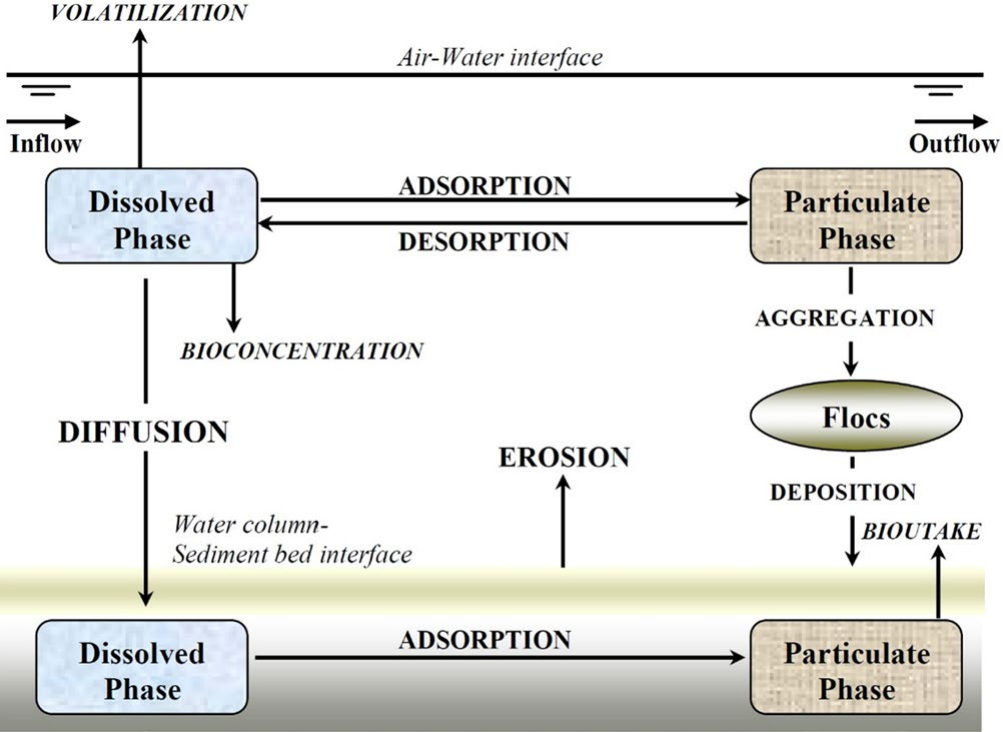
Conceptual model of key processes influencing fate and transport of suspended sediments and associated toxic heavy metals in surface water systems^26^.

Clearly, the quality and availability of water resources are critical to sustaining life and raising the standard of living^1^. Therefore, to avoid pollution and scarcity problems caused by the impact of anthropogenic activities, water resources must be subject to basic sustainability criteria. As a result, numerous studies have been conducted using different methodologies to quantify pollution levels^2^. Water quality models are a computer tool for understanding the dynamics and transport in shallow water habitats (WQM). WQMs allow not only the determination of the behavior and transport of toxic substances, but also the representation of the characteristics and behavior of the relationships within the system using the corresponding analytical prediction capabilities, which are useful for defining approaches and addressing complex problems related to water resources. The review article by Wang et al^3^ provides an overview of computational models for water quality. According to the literature review, the most popular models for water quality simulation in environmental impact assessments are MIKE, EFDC, and Delft 3D.It is important to note that some other models have been developed specifically for research purposes. Examples include numerical modeling of heavy metals^4^, research on water fluxes through vegetation^5^, research on turbulence models^6^, freshwater plumes^7,8^ in river-sea interactions^9^, and numerical assessment of flood risks^10^. The ability to introduce toxic wastes into water bodies without affecting human health or ecosystems is called assimilative capacity and is one of many practical and affordable solutions for water quality management. The assimilation capacity can be used to determine an acceptable pollution concentration that the river can handle to maintain water quality at acceptable levels^11^. Because numerical simulation is considered as the third method after theory and experiment to understand physical and biological problems, numerical modeling is a very helpful tool in environmental research. Because numerical modeling is a very effective analytical tool, we used it to assess water quality and build the modeling^12^. Through simulations, we can also predict the pollutant levels, distributions, and risks^13^. Similarly, numerical modeling can provide a basis and technical assistance for pollution control decision making by using modeled data to predict water quality to some degree^14^ because of the ability of heavy metals to accumulate and persist in the environment and their toxicity, even at low concentrations, they can negatively impact the environment and organisms^15^, making the monitoring of these pollutants critical to the management of river water quality. Several studies have been conducted on the distribution, pollution factors, and treatment options of heavy metal pollution in rivers. Various indicators, such as the enrichment factor and geoaccumulation index^16^, have been used to evaluate the spatial distribution of heavy metals in sediments and the state of heavy metal contamination. In addition, potential sources of heavy metals were investigated and identified. The results of previous research in the field of water quality modeling, for example, by Fanger et al.^17–19^, have been helpful in understanding the dynamics of the transport of contaminants such as heavy metals in rivers. Many studies on the behavior and transport of these pollutants in rivers using hydrologic models have been conducted to reduce heavy metal concentrations. In simulation studies, the transport processes of heavy metals in rivers are typically considered independently by scientists or engineers for different elements and reactions. To achieve better accuracy, Kashefipour^20^ developed a method to predict a fluctuating reaction coefficient for the dissolved concentrations of Pb and Zn using pH and EC, which affect the reaction coefficient in the advection–dispersion equation. For the simulation of dissolved heavy metals, they also presented the best correlations for the reaction coefficients of the dissolved Pb and Cd. Noori et al. showed that metal pollution in the surface sediments of Lake Namak, Iran, was generally low, although the Pb concentration in the southern part of the lake was of concern^21^. Heavy metal pollution studies can provide useful information for the sustainable management of river and reservoir water resources, especially for reservoirs used for drinking water supplies, such as the Sablan Reservoir (SDR) in Iran^21^. The results of the simulations and corresponding measured data agree well. Wu et al^22,23^ used hydro-environmental models to predict the distribution of heavy metal concentrations in the Mersey catchment in the United Kingdom. The accuracy of the models was improved by the three-dimensional advection–diffusion equation and dynamic distribution coefficients, which provided better agreement between the predicted and measured heavy metals. It should be emphasized that most researchers have considered the chemical reactions, the transport pathway of heavy metals for each element in the rivers, and advection–diffusion equations. As the transport of dissolved heavy metals is highly influenced by the flow characteristics, a hydrodynamic model is needed for accurate simulation of the river flow. In addition, due to the influence of the water quality parameters including temperatures, pH, DO, and EC on the transformation process of the dissolved phase of the heavy metals, these parameters also should be simulated. Among the hydrodynamics and water quality models, the Qual2kw hydrodynamics and water quality model have been widely used for simulating flow and water quality parameters in river^24^, However, this model cannot simulate heavy metal transport, so the main objective of the present study was to simulate the transport of dissolved heavy metals in the river and provide a methodology to predict the sorption and desorption processes of dissolved heavy metals such as Pb, Cd, and Zn using the above-mentioned water quality parameters that affect the transformation term of the advection–diffusion equation to improve the modeling accuracy. It should be noted that the choice of the dissolved phase of heavy metals for the simulation is due to uncertainties and the complexity of the particulate phase diffusion process of heavy metals, as well as the direct impact of the dissolved phase of heavy metals on the environment. Also, the selection of heavy metal in the present study has been due to harmful environmental effects of Pb, Cd, Zn and their conventional presence in Sarough River, within the detection limit by the measuring devices, due to the gold mining establishments and industrial factories, the reasons for the increased concentrations of metals in the Zarrineh River may be attributed in particular to anthropogenic and mining activities^25^. In the present paper, the development details of a model for the prediction of dissolved Pb, Cd, and Zn concentrations and the application of the model to the Sarough River are presented. Figure 1 shows the key processes of the conceptual model for heavy metals in rivers.

The calibration of models requires a substantial amount of data, which is rarely feasible, particularly where heavy metal poisoning of rivers is a common occurrence, as in the case of the Sarouq River in Iran. Heavy metal pollution from numerous enterprises has badly harmed the Sarouq River^27,28^. The lack of broad pollution monitoring and lax enforcement of environmental rules has made it difficult to gather enough observational data on contaminants in stream water and sediment to study environmental countermeasures and explore strategies for contamination countermeasures in developing countries, especially where few observational data are available. This study aimed to evaluate the effectiveness of hydrological models in predicting the movement of heavy metals in rivers. Therefore, the use of the Qual2kw model and VBA coding to evaluate contamination by various heavy metals in the Sarouq River was described. Because little observational data are available due to information availability problems, the model was used to assess contamination. Three industrially produced heavy metal concentrations Pb, Cd, and Zn were simulated in the river network.

## Material and methods

### Description of the study area

**Figure 2:**
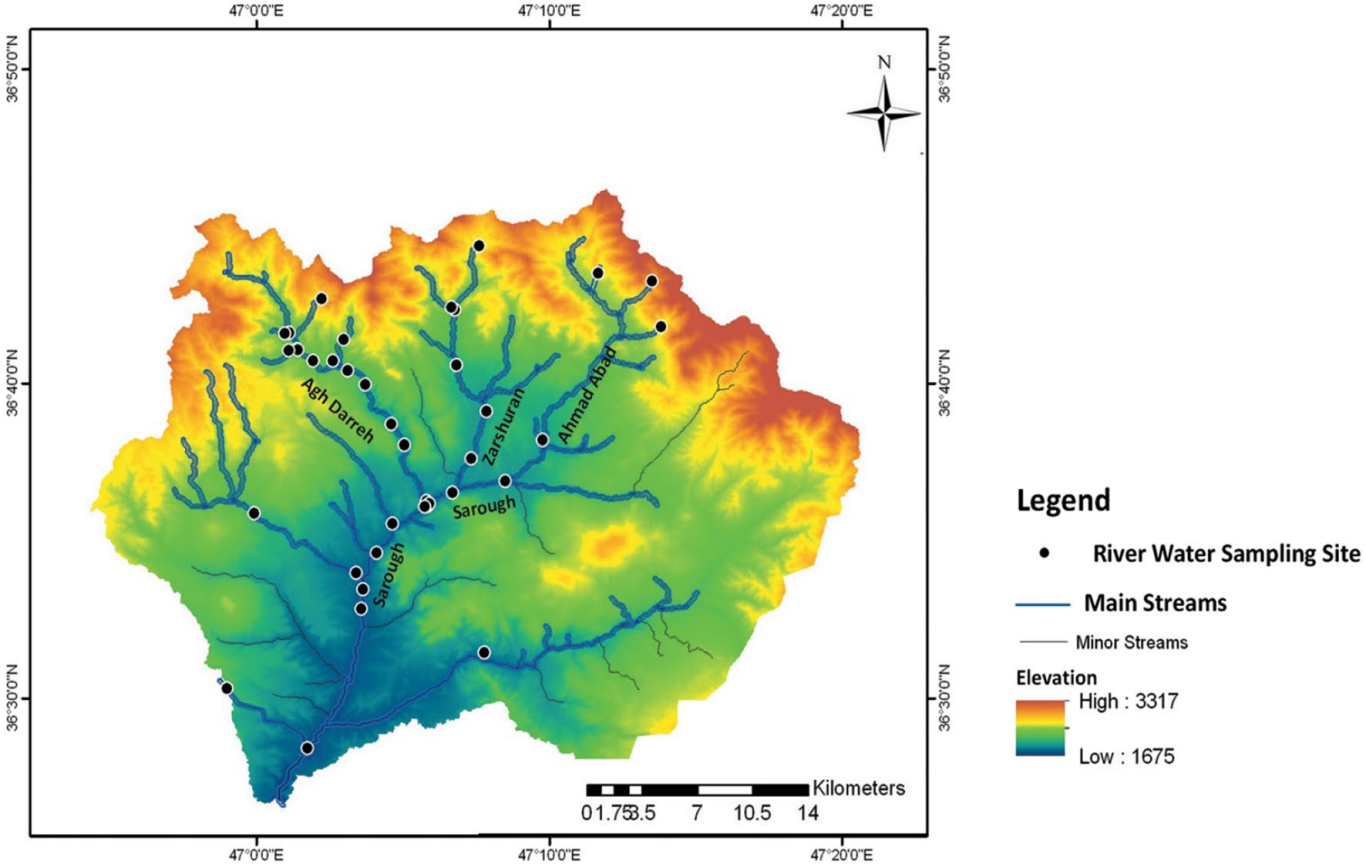
Location map of main streams in Sarouq watershed and sampling sites. (This figure was created in the Quantum GIS, version 3.12.2).

The Sarouq watershed is located 10 km north of Takab, West Azerbaijan province, NW Iran, as Fig. 2 is showed the location of Sarouq watershed. The region covers an area of approximately 913 km^2^. The Sarouq River (length 40 km, average annual flow 3.31 m^3^ s^−1^) is the main permanent river which passes through the watershed^29^. Zarshuran, Aq-Darreh and Ahmad Abad streams are the main tributaries of Sarouq River which supply the agriculture and drinking water for several populated areas. The catchment area of the Sarouq River is generally covered by mountains, particularly in the northern parts.

### Qual2kw model

The American Natural Assurance Organization planned Qual2k models. It is one of the most notable instruments for reproducing water quality, with key advantages of flexibility, availability, and convenience^30^. The Qual2k model has the potential for the reenactment of various water quality boundaries in wet and dry seasons, as well as lengthy and short waterways, and can likewise run with low measures of information. QUAL2Kw can reproduce a number of water quality boundaries including temperature, pH, carbonaceous biochemical interest, dregs oxygen interest, disintegrated oxygen, natural nitrogen, alkali nitrogen, nitrite and nitrate nitrogen, natural phosphorus, inorganic phosphorus, absolute nitrogen and phosphorus^31^. Zhang et al. considered the nature of the Taihu Lake Bowl utilizing QUAL2K model and presumed that the water nature of this lake was the consequence of wastewater and profluent release in Hongqi Stream which streams in Taihu Lake^32^. Giraldo et al. displayed the 50 km of the Aburra-Medellin Stream utilizing the Qual2kw model. An aligned model with three situations (short, medium, and long durations) was studied. The outcomes showed beneficial effects of Bello's sewage treatment on equilibrium, oxygen and nitrogen^33^.

### Model formulation

The advection–diffusion equation governing one-dimensional flow pollution indicators can usually be simulated using the mass balance equation using numerical methods. The dynamic equation of mass balance in the form of an advection–diffusion relationship for a one-dimensional state is the basis of water quality modeling. Before discussing the modeling of pollution parameters using ADE, in this section, some key concepts related to the modeling of water pollution using ADE are introduced. The concentration of a substance in a specific place of the system is constantly changes because of the physical processes of movement and dispersion that transfer fluid components from one place to another. The total amount of substance in the closed system was constant. Unless it is altered by physical, chemical, or biological processes. Using Fick's method for turbulent mass flux, the one-dimensional advection–diffusion Eq. (1) and mass balance can be written as follows:

1 $$\frac{\partial c}{\partial t}=\frac{\partial \left({AD}_{L}\frac{\partial c}{\partial x}\right)}{A\partial x}-\frac{\partial \left(A\bar{u}c\right)}{A\partial x}+\frac{dc}{dt}+\frac{s}{V}$$

where C is the concentration of the pollutant (mg/L), t is time (s), A is the cross-sectional area of the element perpendicular to the flow (m^2^), D_L_ is the dispersion coefficient (m^2^/s), x is the length of the river (m), u is the average speed of the flow (m/s), s is the sink or source (mg), and V is the volume of the element (m^3^). The expression $\frac{dc}{dt}$ expresses the changes in the concentration of the polluting substance under environmental processes, and for each substance, it differs according to the production or consumption processes. In addition, the term on the, $\frac{\partial c}{\partial t}$, expresses the concentration gradient.

The model uses three hydraulic balance equations, heat balance equations, and mass balance equations to calculate flow rate, heat, and concentration of qualitative parameters.

2 $${\left[{\mathrm{H}}^{+}\right]}^{4}+\left({\mathrm{K}}_{1}+\mathrm{Alk}\right){\left[{\mathrm{H}}^{+}\right]}^{3}+\left({\mathrm{K}}_{1}{\mathrm{K}}_{2}+{\mathrm{AlkK}}_{1}-{\mathrm{K}}_{\mathrm{w}}-{\mathrm{K}}_{1\mathrm{cr}}\right){\left[{\mathrm{H}}^{+}\right]}^{2}+\left({\mathrm{AlkK}}_{1}{\mathrm{K}}_{2}-{\mathrm{K}}_{1}{\mathrm{K}}_{\mathrm{w}}-{2\mathrm{K}}_{1}{\mathrm{K}}_{2}{\mathrm{C}}_{\mathrm{T}}\right)\left[{\mathrm{H}}^{+}\right]-{2\mathrm{K}}_{1}{\mathrm{K}}_{2}{\mathrm{K}}_{\mathrm{w}}=0$$

where $\left[{\mathrm{H}}^{+}\right]$ is hydrogen ion concentration (in moles), Alk is alkalinity, ${\mathrm{C}}_{\mathrm{T}}$ is total inorganic carbon, and ${\mathrm{K}}_{1},$ ${\mathrm{K}}_{2}$ and ${\mathrm{K}}_{w}$ are constant numbers at known temperature, in terms of carbonate [CO_3_^2−^] , bicarbonate [HCO_3_^−^] $,\left[{\mathrm{OH}}^{-}\right]$ and $\left[{\mathrm{H}}^{+}\right]$ are obtained from the following formulas and finally the pH parameter is calculated from the formula

3 $${\mathrm{pK}}_{\mathrm{w}}=\frac{4787.3}{{\mathrm{T}}_{\mathrm{a}}}+7.1321 \,{\mathrm{log}}_{10}\,({\mathrm{T}}_{\mathrm{a}})+0.010365{\mathrm{T}}_{\mathrm{a}}-22.80$$

4 $${\text{log}}{\mathrm{K}}_{1}=-\text{356.3094}-0.06091964{\mathrm{T}}_{\mathrm{a}}+\frac{21834.37}{{\mathrm{T}}_{\mathrm{a}}}+126.8339\,{\mathrm{logT}}_{\mathrm{a}}-\frac{1684915}{{\mathrm{T}}_{\mathrm{a}}^{2}}$$

5 $${\text{log}}{\mathrm{K}}_{2}=-107.8871-0.03252849{\mathrm{T}}_{\mathrm{a}}+\frac{5151.79}{{\mathrm{T}}_{\mathrm{a}}}+38.92561\,{\mathrm{logT}}_{\mathrm{a}}-\frac{\mathrm{563,713.9}}{{\mathrm{T}}_{\mathrm{a}}^{2}}$$

6 $$\mathrm{pH}=-{\mathrm{log}}_{10}\left[{\mathrm{H}}^{+}\right]$$

### The method of solving equations

With the aid of one of these three relationships chosen by the user in accordance with the requirements of the research area, this model first calculates the flow rate of each element using the flow balance equation, and then calculates the parameters of flow speed and water height. The model carries out the Euler or Ranguta method numerical dissolution of the advection–diffusion equations. Ranguta’s approach is more accurate than Euler's method, but Euler’s method is quicker, therefore the user can select one of these ways depending on the needs of the project. In addition, the model provides the user with three methods to solve the pH equation,:Brent’s method, Newton–Raphson’s method, and the two-part method. Brent's method is accurate but more time-consuming, Newton–Raphson’s method is faster than the other two methods but with lower accuracy, and the two-part method is used when the other two methods cannot be used. Four categories can be derived from the data needed for modeling:

#### Geometrical data

Such as the cross-section data at the beginning and end of intervals, the diffusion coefficient, the length of the intervals, the height level at the beginning and end of each interval, and their geographic locations.

#### Hydraulic data

Comprises flow rate, speed, roughness coefficient, longitudinal slope in each span, cross-section width in each span, and lateral slope of trapezoidal sections, among others.

#### Meteorological details

Such as wind speed, air temperature, dew point temperature, amount of sunlight, and cloud cover.

#### Quantitative and qualitative information

Including qualitative data values, along with coefficients corresponding to each process in each selected branch of the river, and information on point sources of pollutants along the route, which have already been sampled.

### Calibrating the model

Calibration must be performed to adjust the model to reality after the input data has been entered and the model's requisite states for simulation have been established. The Qual2kw model can be calibrated in two ways. In the first method the effective coefficients of the model were manually determined through a trial-and-error process. The second approach uses an automatic calibration included in the model which uses a genetic algorithm to automatically carry out the calibration. The genetic algorithm was used to determine the best values for the conventional coefficients of the model variables. The explanation is divided into two sections; the first section describes the genetic algorithm, and the second describes the genetic algorithm of the Qual2kw model.

### Genetic algorithm

John Holland originally suggested the use of genetic algorithms as effective search and optimization tools in his doctoral thesis in 1974. David Goldberg and other of his students went on to develop it, and research on this potent tool is still going strong today. The primary principle of this approach is that, after considering a set of answers at random, new answers are generated from one generation to the next using the three fundamental operators of selection, combination (or exchange), and mutation. This operation performed to ensure the new chromosomes contain the appropriate and good parts of the previous chromosomes. The closer the value of these functions is to zero, the better the fit. Determining the best value for Pm also requires testing or understanding the problem. There are different methods to choose from, including the residual, uniform, competitive and roulette wheel methods. The only method built into the genetic algorithm in the Oual2kw model for selection is the roulette-wheel method. Raising the proportion of desired chromosomes in the population. If the algorithm has not yet reached the stopping condition after creating a new population using the three aforementioned operators, then the prior population should be correctly replaced with the current population. The PIKAYA algorithm selection operator includes the following three techniques:

The first involves removing parents and replacing them with their children. The second method involves selecting parents with the worst fitness values. The third method involves randomly selecting parents from a population to replace them. After determining the settings of the genetic algorithm for automatic calibration, the fitting function of this algorithm must be selected to the model. PIKAIA is a subprogram of the FORTRAN77 programming language. This subprogram deals with maximizing the value of the objective function.

7 $$RNRMSE = \left[ {\sum\limits_{i = 1}^{q} {w_{i} } } \right]\left[ {\sum\limits_{i = 1}^{q} {\frac{1}{{w_{i} }}\left[ {\frac{{\frac{{\sum\limits_{j = 1}^{m} {O{}_{ij}} }}{m}}}{{\left[ {\frac{{\sum\limits_{j = 1}^{m} {\left( {P_{ij} - O_{ij} } \right)^{2} } }}{m}} \right]^{\frac{1}{2}} }}} \right]} } \right]$$

When presenting the formula's findings, the numbers in (7) are set back to their original values to make comparisons and inferences easier. where q is the number of selected qualitative parameters, w_i_ is the weight factor for the ith state variable, m is the number of observation data points along the river, O_ij_ is the observation data value of the ith state variable at the jth station of the river, and P_ij_ is the data value predicted by the software for the state variable i at the location of the jth station of the river. In this study, NSFWQI method weight coefficients were used to determine the value of the weight factor (w_i_) of the qualitative parameters. Additionally, in this study, the calibration outcomes were evaluated using two absolute average errors and the root mean square error, using the following formulas (comparison of model output graphs).

8 $$AME=\left|\frac{{X}_{m}-{X}_{o}}{n}\right|$$

9 $$RMSE=\sqrt{\frac{\sum {({X}_{m}-{X}_{o})}^{2}}{n}}$$

where n is the number of observational data points, X_o_ is the value of the observational data, and X_m_ is the value of the data produced by the model.

### Heavy metals simulation

**Figure 3:**
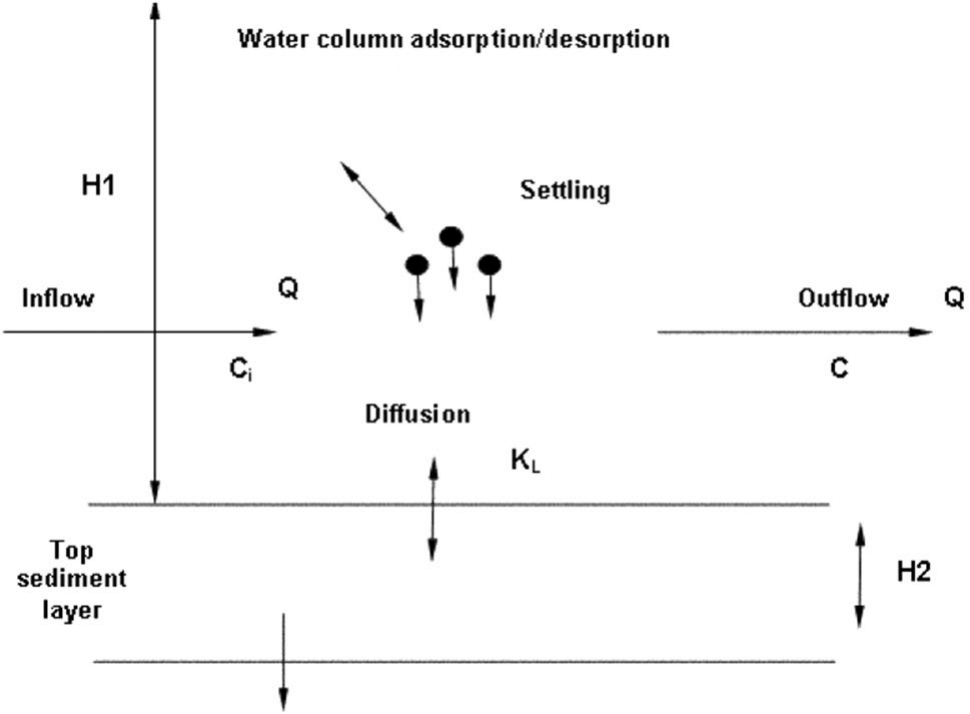
Heavy metals are transported and dispersed throughout the river system^34^.

Understanding the behavior of heavy metals in the system is important for modeling their transport of heavy metals in river systems. Figure 2 shows that heavy metals exist in the river in two forms, particulate and dissolved, where the particulate phase is absorbed into the bed sediments and the dissolved phase is found in the water column^34^. Owing to their complex nature, the concentration of heavy metals is affected by a variety of factors, including temperature, salinity, pH, dissolved solids, dissolved oxygen, and electrical conductivity. The behavior of heavy metals is strongly influenced by the absorption of organic and inorganic substances. They can exit the dissolved state or re-dissolve in the water column, depending on the geographical and temporal circumstances. Researchers have used a set reaction coefficient as shown^34^ in Fig. 3 with time to forecast the fate and transit of heavy metals; however, field data show that this coefficient can change depending on the pH, salinity, temperature, or even other chemicals and other substances. There are differences in the hydraulic properties of rivers.

A simulation of the heavy metals Pb, Cd, and Zn was performed in this study. The general procedure is as follows. First, a module named the heavy metal module was added using VBA coding. According to, the equations for simulating the dissolved phase of heavy metals are entered in (11).

The dissolved phase of heavy metals in a stable and one-dimensional system is transferred according to Eq. (10):

10 $$\frac{\partial CA}{\partial t}+\frac{\partial CQ}{\partial x}-\frac{\partial }{\partial x}\left[A{D}_{x}\frac{\partial C}{\partial x}\right]={S}_{0}^{d}+{S}_{t}^{d}$$

11 $${S}_{0}^{d}=\frac{{Q}_{L}{C}_{L}}{\Delta x}$$

12 $${S}_{t}^{d}=-k{C}^{m}A$$

where C is the pollutant concentration (mg/L), t is the time (s), A is the cross-sectional area of the element perpendicular to the flow (m^2^), D_x_ is the diffusion coefficient^35,36^ (m^2^/s), x is the river length (m), Q is the flow rate (m/s), ${\mathrm{S}}_{0}^{\mathrm{d}}$ is the expression of the source or external well of dissolved heavy metals (mg), and C_L_ is the concentration of heavy metals entering and leaving the river. The reactivity of heavy metals (mg) is expressed as ${S}_{t}^{d}$, and the reaction coefficient is k. The reaction order is m in this study, it is considered equal to one). In the second stage, the initial conditions sheet was used to introduce the data linked to the concentration of heavy metals in the sample stations. The reactivity coefficient of the relevant heavy metal was entered by assuming a number in the third step's sheet related to the automatic calibration of the model. For automatic calibration via the genetic algorithm, the range of the reaction coefficient of the desired heavy metal dissolved phase must be entered into the model. Following program development and calibration, the genetic algorithm uses the concentrations obtained from the model to determine the values of k. On the other hand, the formula used in this study considers the reactivity coefficient of the dissolved phase of heavy metals as a function of pH, temperature, dissolved oxygen, and EC (13). Two relationships considered to present the relationship between the qualitative factors and the reactivity coefficient of the heavy metal dissolved phase in accordance with formulas (14) and (15).

13 $$\mathrm{k}=\mathrm{f}\left(\mathrm{pH},\mathrm{DO},\mathrm{EC},\mathrm{T}\right)$$

14 $$\mathrm{k}={\mathrm{a}}_{1}\mathrm{T}+{\mathrm{a}}_{2}\mathrm{DO}+{\mathrm{a}}_{3}\mathrm{pH}+{\mathrm{a}}_{4}\mathrm{EC}+{\mathrm{a}}_{5}$$

15 $$\mathrm{k}={\mathrm{a}}_{1}\,\mathrm{ln T}+{\mathrm{a}}_{2}\,\mathrm{ln DO}+{\mathrm{a}}_{3}\,\mathrm{ln pH}+{\mathrm{a}}_{4}\,\mathrm{ln EC}+{\mathrm{a}}_{5}$$

Coefficients a_1_, a_2_, a_3_, a_4_, and a_5_ in Eqs. (14) and (15) are constants. K, temperature, dissolved oxygen, pH, and EC data were used to calculate the constant coefficients of the k equations. After finding the constant coefficients for the k equations, we go back to the Qual2kw model and add the reaction coefficient to it twice using the formulas (14) and (15), respectively, in the additional simulation module. The model is then run again, but this time manual calibration is used to reduce model error when using observational data. Sarouq River simulation in both quantitative and qualitative terms Using version 6.0 of the Qual2kw program is preferable to version 5.1, which only considers permanent mode for modelling, because dynamic river modelling is one of the characteristics of version 6.0 (the most recent updated version). It appears improved. Version 6.0 of this model, however, is more of a pseudo-dynamic model because the identical equations as version 5.1 are calculated separately for each of the 365 days of the year and there is no continuity in the data connected to those 365 days. It matters. For this reason, the Sarouq River was simulated using the Qualitative model Qual2kw version 5.1. Because the Sarouq River is dry during the summer, only three seasons spring, autumn, and winter were taken into consideration for the duration of the quantitative and qualitative simulation and calibration period.

### Establishing the model’s boundary conditions

**Figure 4:**
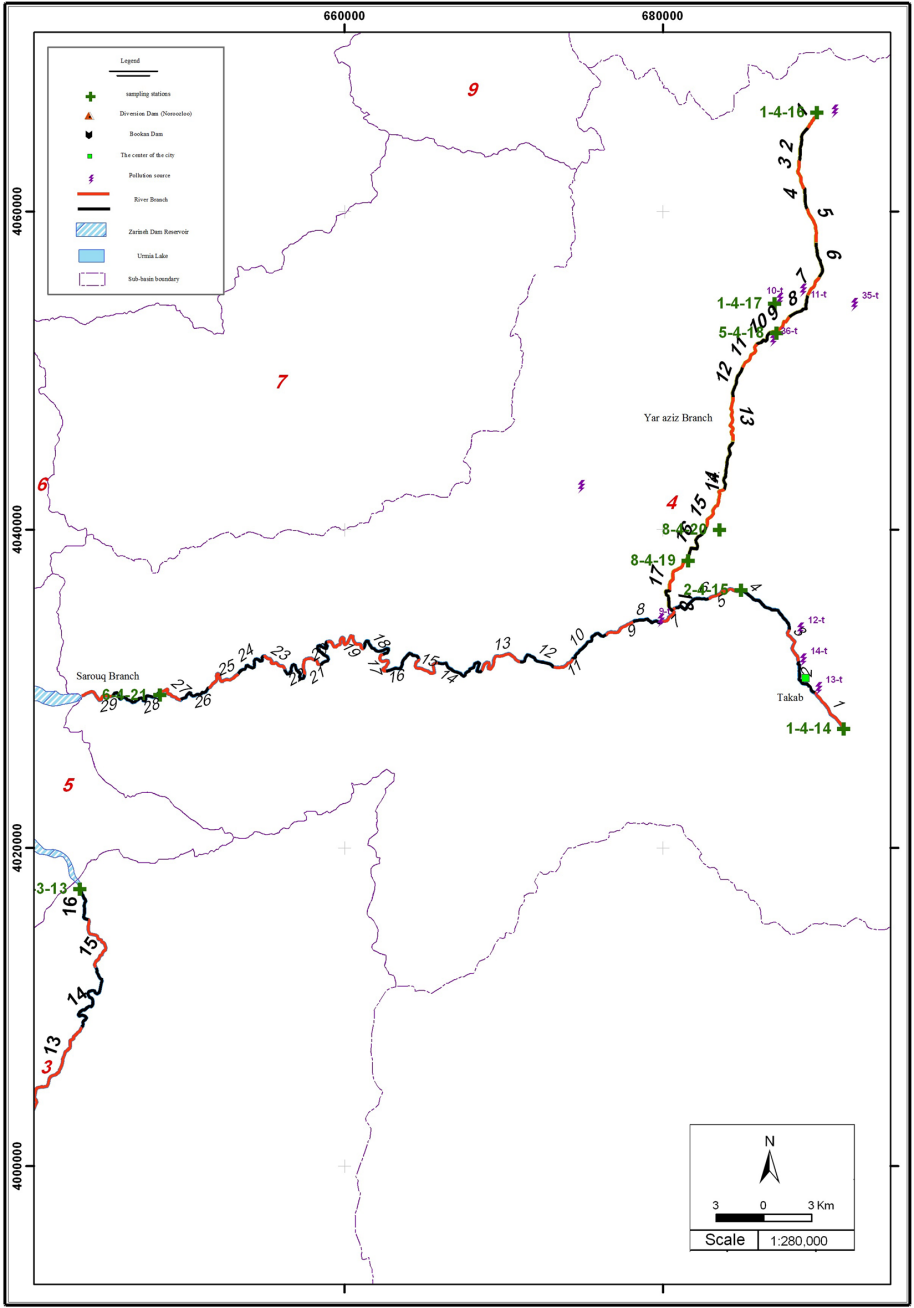
Location of sampling stations. (This figure was created in the Quantum GIS, version 3.12.2).

The flow rate and quality parameters relating to the upstream and downstream stations of the river were recorded in this sheet. Figure 4 shows that the Sarouq River has three sampling stations along its course, with stations 1–4–14 and 6–4–21 situated alternately upstream and downstream of it, respectively. The study region in Table 1, is presented in the model in accordance with Fig. 4 in the boundary conditions sheet of the specifications of these stations, is where these stations are located.

**Table 1:** Specifications of sampling stations.

Station	The distance from the station to the end (km)	Specification of station	Province
1–4–14	78.15	Above the city of Takab	West Azerbaijan
2–4–15	66.03	Sarouq downstream of Takab city	
6–4–21	6.61	Sarouq before entering Bukan dam	

According to Table 2 Yar Aziz river is one of the branches that join Sarouq River, and the quantitative and qualitative information related to this branch has also been entered into the model as a point pollutant.The Table 3 Shows the 29 reaches considered for modeling.

**Table 2:** Specifications of the input reaches to the main reach.

Station	The distance from the station to the end (km)	specification of station	Province
8–4–19	61.2	Yar aziz river	West Azerbaijan

For the Sarouq River, with a length of 78.15 km, 29 intervals with variable intervals were considered. Among the points that can be used as the beginning and end of the interval are those with a sudden change in the concentration of dissolved oxygen. This parameter wasbeen calculated using the river information layers in a GIS environment. Data on the hydraulic and geometrical conditions of the Sarouq River in Iran, as well as the location of sampling stations, were considered. The geographical coordinates of the beginning and end of the sampling intervals were determined using Google Earth Pro 7.3.6.9345 to provide finally the Fig. 4 segmentation and interval reaches by the Quantum GIS, version 3.12.2.

**Table 3:** The length of Sarouq reach.

Number	Length	Cumulative length
1	2.92	78.15
2	2.63	75.24
3	2.39	72.60
4	4.17	70.21
5	2.31	66.04
6	2.52	63.73
7	1.49	61.21
8	1.95	59.72
9	2.05	57.77
10	3.05	55.72
11	4	52.67
12	2.65	51.22
13	3.92	48.57
14	4.37	44.65
15	2.73	40.28
16	2.82	37.55
17	1.98	34.72
18	2.88	32.74
19	4.09	29.86
20	2.87	25.77
21	2.16	22.91
22	2.59	20.74
23	1.89	18.15
24	2.70	16.26
25	2.98	13.56
26	2.26	10.57
27	1.70	8.32
28	4.13	6.62
29	2.49	2.49

### Point sources of pollution importation

Table 4 shows that point pollution from the sewage of Takab City, the Omid Cardboard Factory, and sand washing enters the river at 4 locations along its course.

**Table 4:** Characteristics of polluting sources along the Sarouq reach (km).

Station	Distance to the end	Pollution source	City	Province
13-t	75.24	Takab wastewater	Takab	Western Azerbaijan
14-t	72.6	Takab wastewater	Takab	
12-t	70.21	Omid Paper cartooning	Takab	
9-t	59.72	sand wash	Takab	

### Model calibration

**Figure 5:**
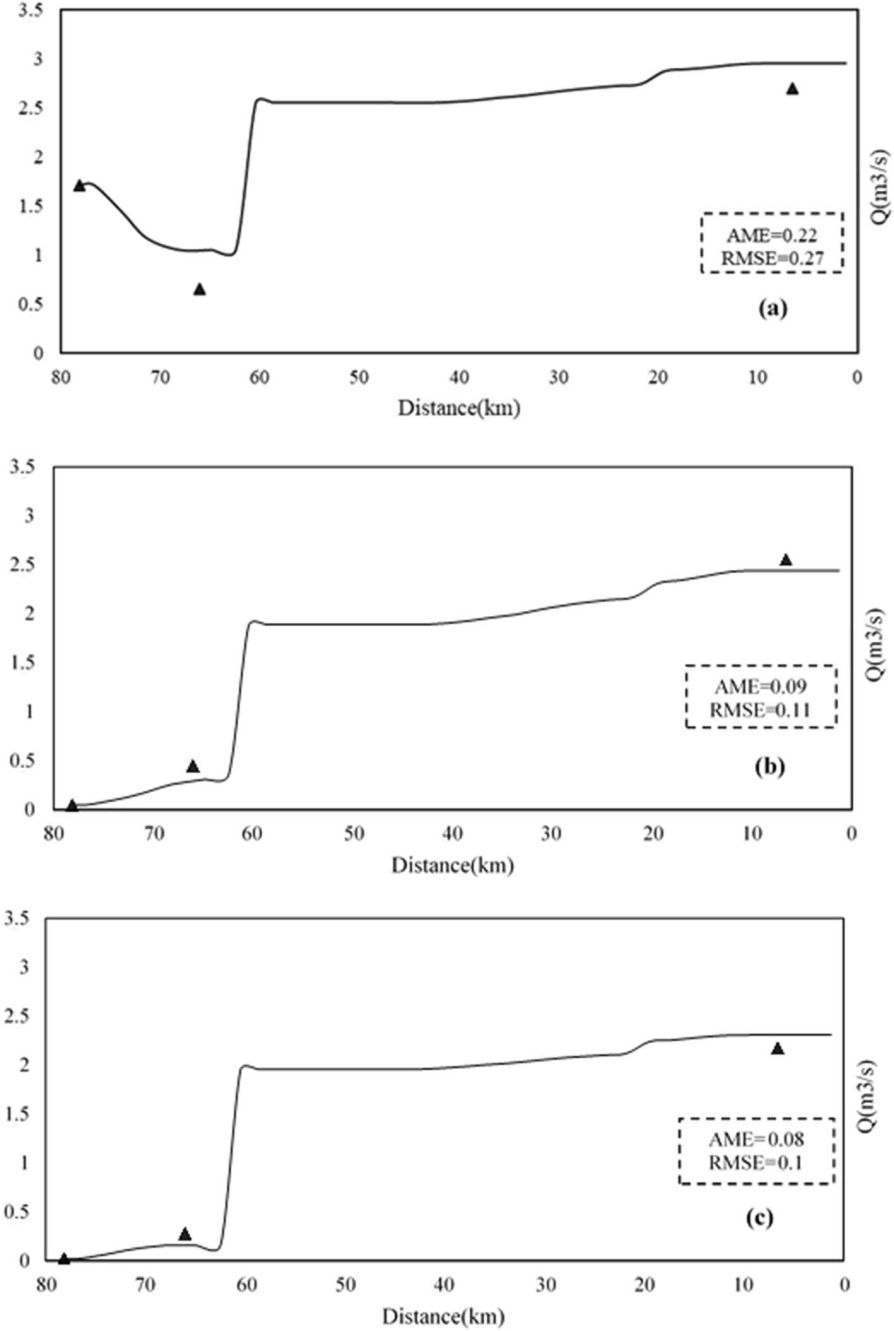
Discharge of Sarouq River in (**a**) spring, (**b**) autumn, (**c**) winter after calibration.

**Figure 6:**
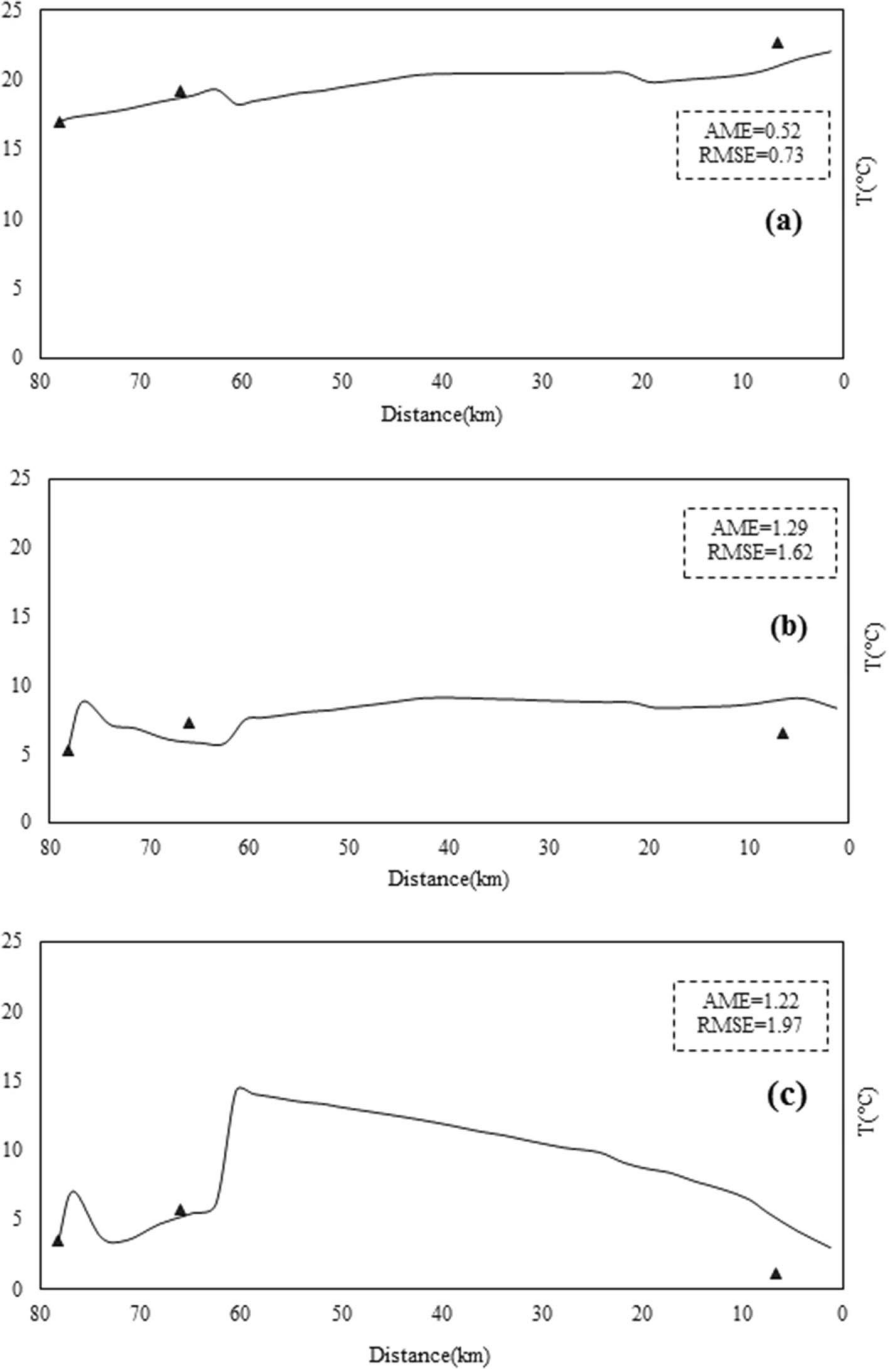
Total Temperature of Sarouq River in (**a**) spring, (**b**) autumn, (**c**) winter after calibration.

**Figure 7:**
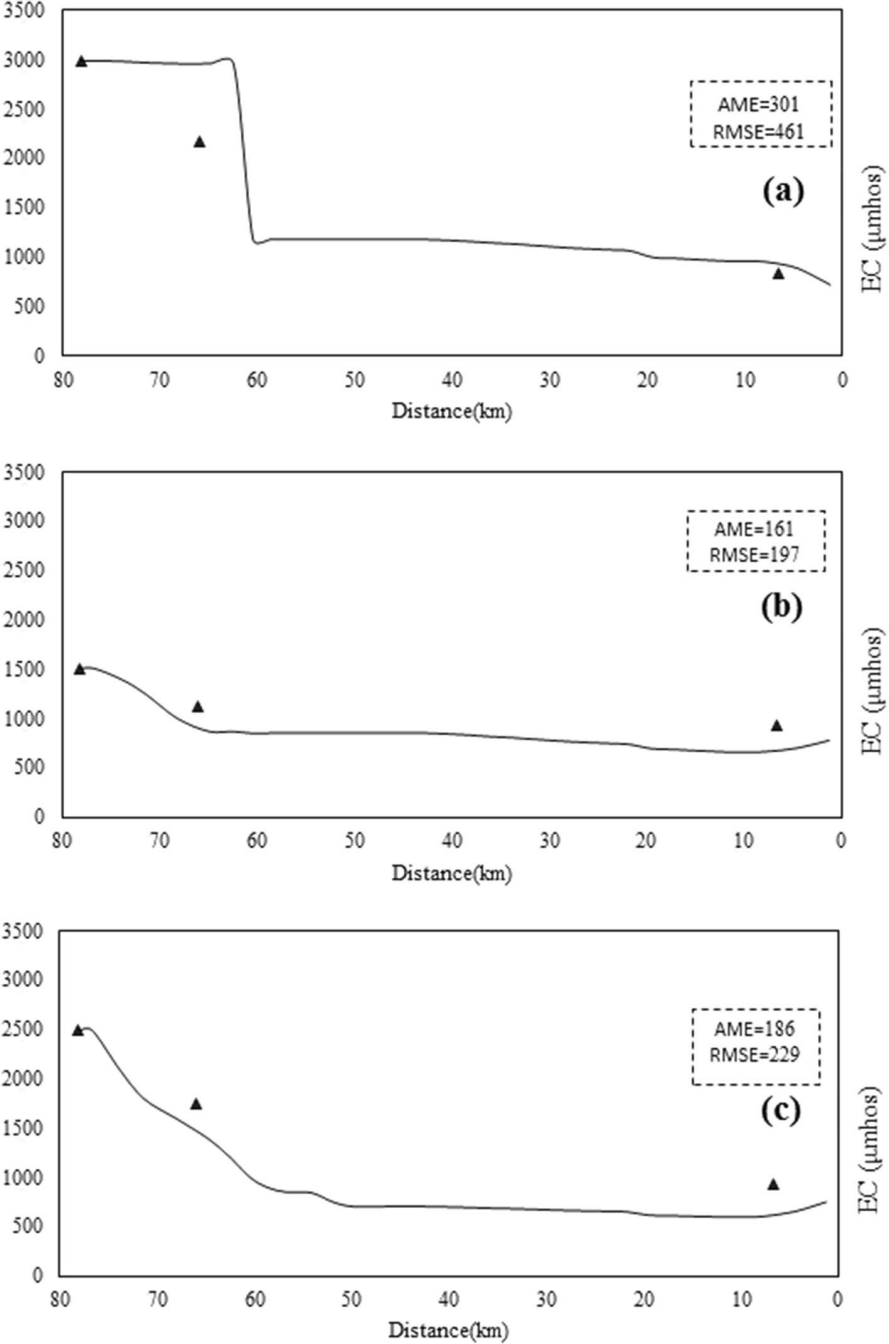
Total electric conductivity of Sarouq River in (**a**) spring, (**b**) autumn, (**c**) winter after calibration.

**Figure 8:**
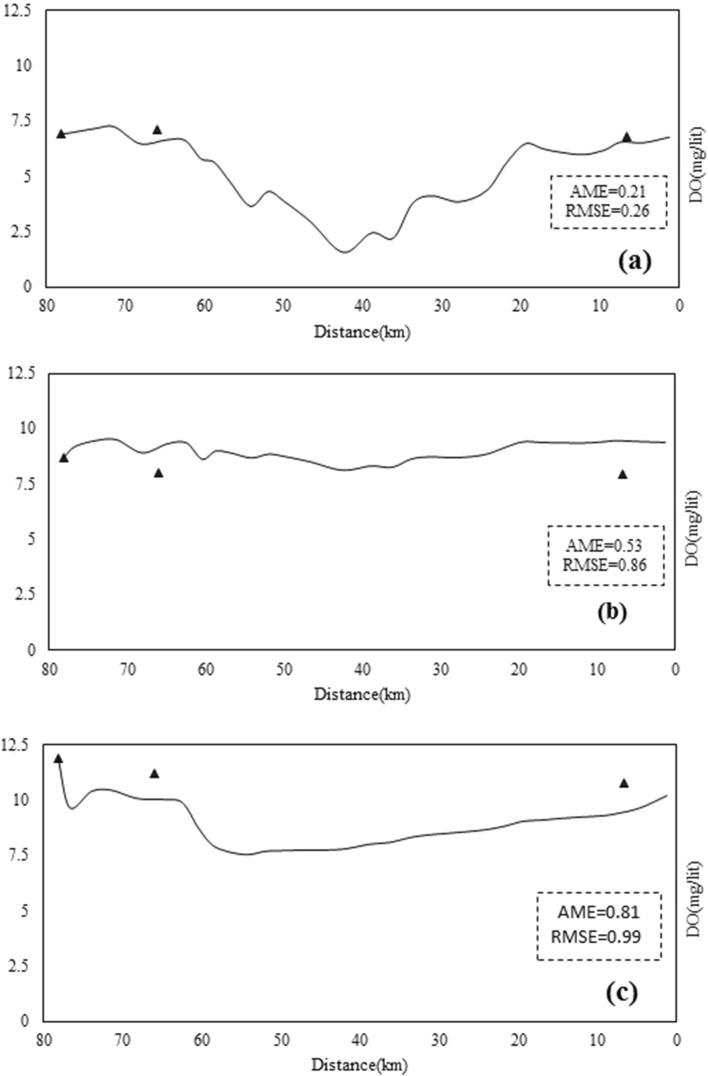
Total dissolved oxygen content of Sarouq River in (**a**) spring, (**b**) autumn, (**c**) winter after calibration.

**Figure 9:**
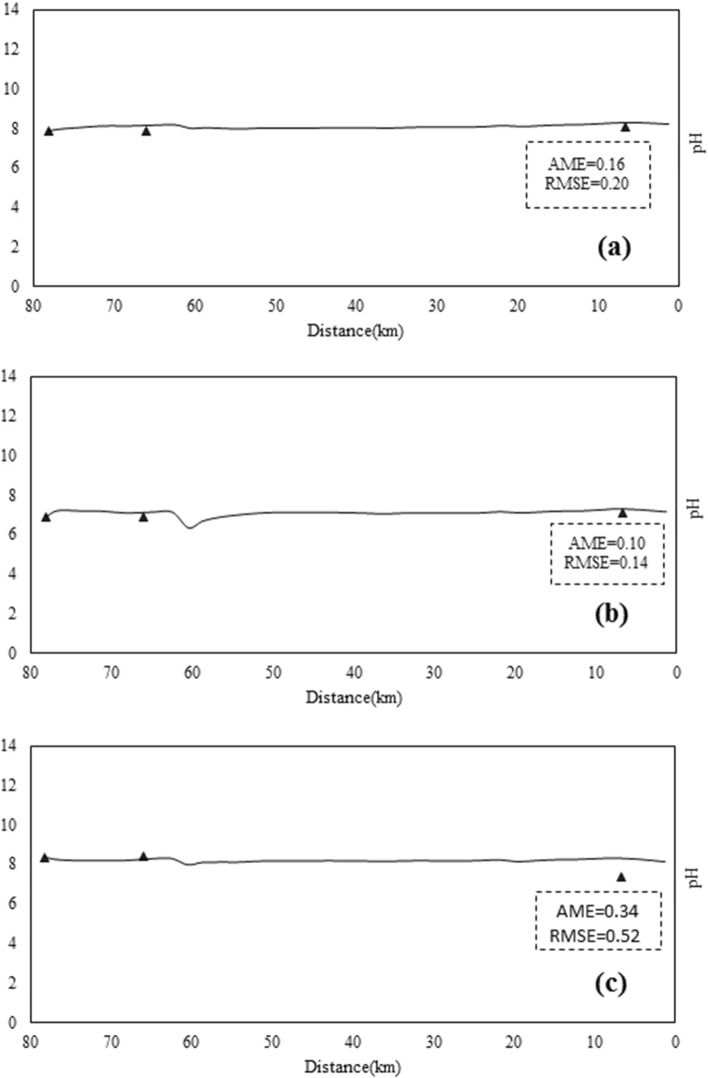
Total pH of Sarouq River in (**a**) spring, (**b**) autumn, (**c**) winter after calibration.

Therefore, the model of the Sarouq River is calibrated only for the qualitative parameters of temperature, DO, pH, and EC. The goal of this research is to present the relationship between the reactivity coefficient of the dissolved phase of the heavy metals lead, cadmium, and zinc. The Qual2kw model can be calibrated manually or automatically, depending on the situation. In this study, the automatic model calibration approach, which employs a genetic algorithm for this task, was used to calibrate the aforementioned qualitative parameters. Figures 5, 6, 7, 8 and 9 show the results of quantitative and qualitative calibration of Sarouq River with Qual2kw model. The duration of running the genetic algorithm for calibration lasted 2500 times, 14 h for each season. River discharge in spring, autumn and winter has been simulated by the model with a very low error. Figure 5 Show the simulated temperature of Sarouq River. According to the graphs, the temperature of the river in autumn and winter is much lower than that of spring. The graphs also show the amount of dissolved oxygen in the river.

## Results

### Developed Qual2kw model for simulating heavy metals

To model the concentration of the heavy metals in the dissolved phase using code in the Qual2kw software’s VBA environment, the heavy metal module was added. Then, an interval was established for automatic calibration based on the assumption that the coefficients of the k equations are numerical according to the concentration of heavy metal, temperature, dissolved oxygen, pH, and EC in various seasons (genetic algorithm method). Next, using the evolutionary algorithm's fitting function, the model error in simulating the concentration of heavy metal dissolved phase with observational data was reduced. The inverse function of the root mean square of the normalized error was chosen as the fitting function introduced to the genetic algorithm and was introduced to the model. K values were calculated, taken into consideration, and entered into linear and logarithmic equations after the adoption of automatic calibration to calculate the concentration of heavy metals with the dissolved phase concentration of heavy metals. Given that there are five unknown coefficients in k equations, five values of k must be placed into the equations; two of these numbers were chosen for the spring, two for the autumn, and one for the winter. The constant coefficients of the equations were therefore determined using Tables 5 and 6 the values of k, temperature, dissolved oxygen, pH, and EC. It is important to note that the units of DO, T, EC, and k_i_ in these tables are, respectively, mg/L, °C, umhos, and 1/day (i: is the name of the metal).

**Table 5:** Linear equations of reaction coefficient of dissolved phase of heavy metals.

Number of equation	Heavy metal	Equation
16	Pb	${k}_{pb}=0.00509\mathrm{T}+0.03508\mathrm{DO}-0.2643\mathrm{pH}+0.00014\mathrm{EC}+1.44994$
17	Cd	${\mathrm{k}}_{cd}=-0.08374\mathrm{T}-0.53401\mathrm{DO}+3.82731\mathrm{pH}-0.00193\mathrm{EC}-20.5194$
18	Zn	${\mathrm{k}}_{zn}=0.00172\mathrm{T}+0.01256\mathrm{DO}-0.09666\mathrm{pH}+0.00005\mathrm{EC}+0.54081$

**Table 6:** Logarithmic equations of the reaction coefficient of heavy metal dissolved phase.

Number of equation	Heavy metal	Equation
19	Pb	${\mathrm{k}}_{pb}=-0.01268\,\mathrm{ln T}-0.05859\,\mathrm{ln DO}+0.23847\,\mathrm{lnpH}-0.02424\mathrm{ln EC}-0.14389$
20	Cd	${\mathrm{k}}_{cd}=0.07045\,\mathrm{ln T}+0.49898\,\mathrm{ln DO}-2.83921\,\mathrm{lnpH}+0.2832\mathrm{ln EC}+2.50094$
21	Zn	${\mathrm{k}}_{zn}=-0.00659\,\mathrm{ln T}-0.02739\,\mathrm{ln DO}+0.10763\,\mathrm{ln pH}-0.01018\,\mathrm{ln EC}-0.05939$

**Figure 10:**
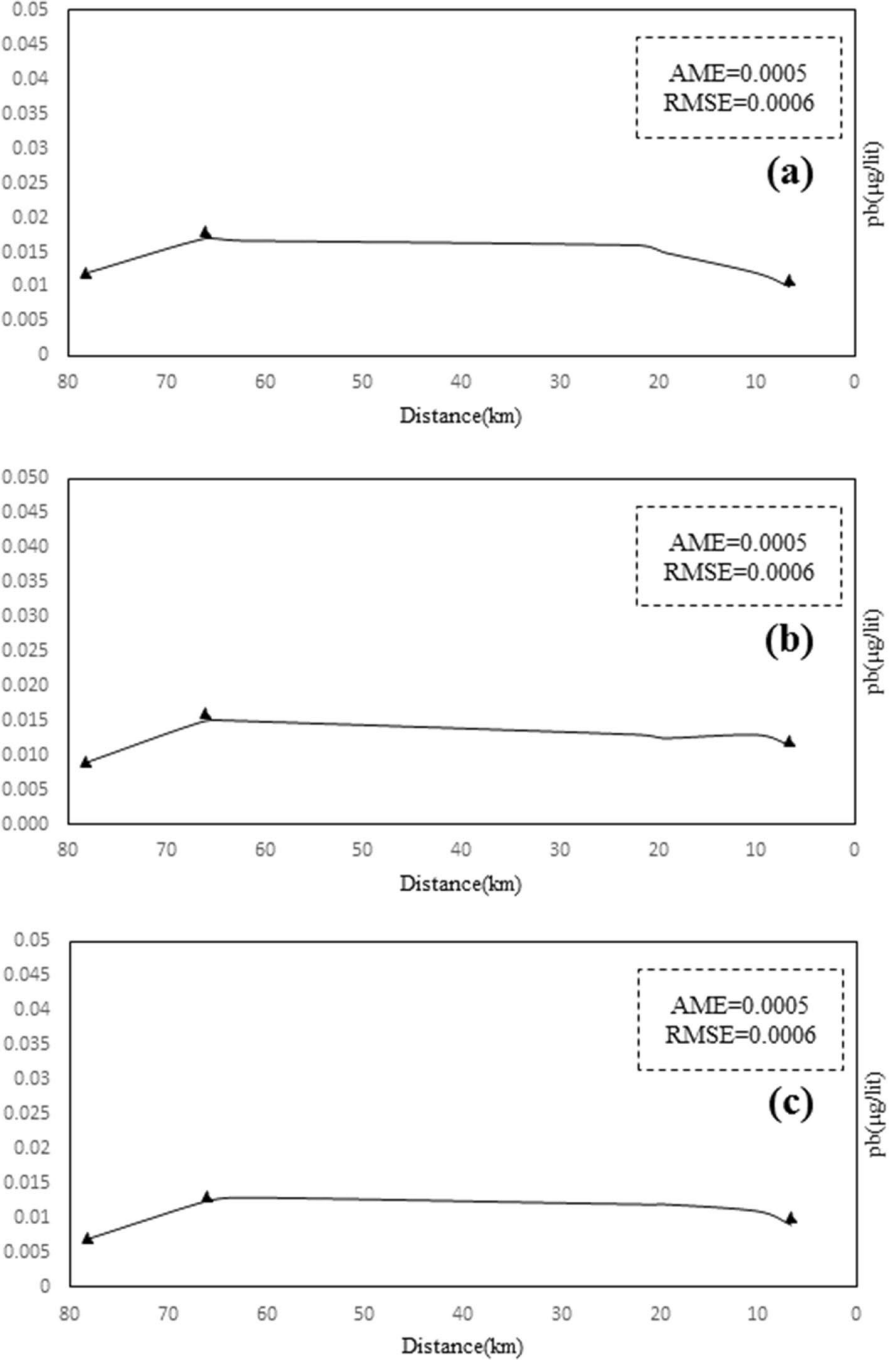
Total lead concentration of Sarouq River in (**a**) spring, (**b**) autumn, (**c**) winter in the developed Qual2kw model.

**Figure 11:**
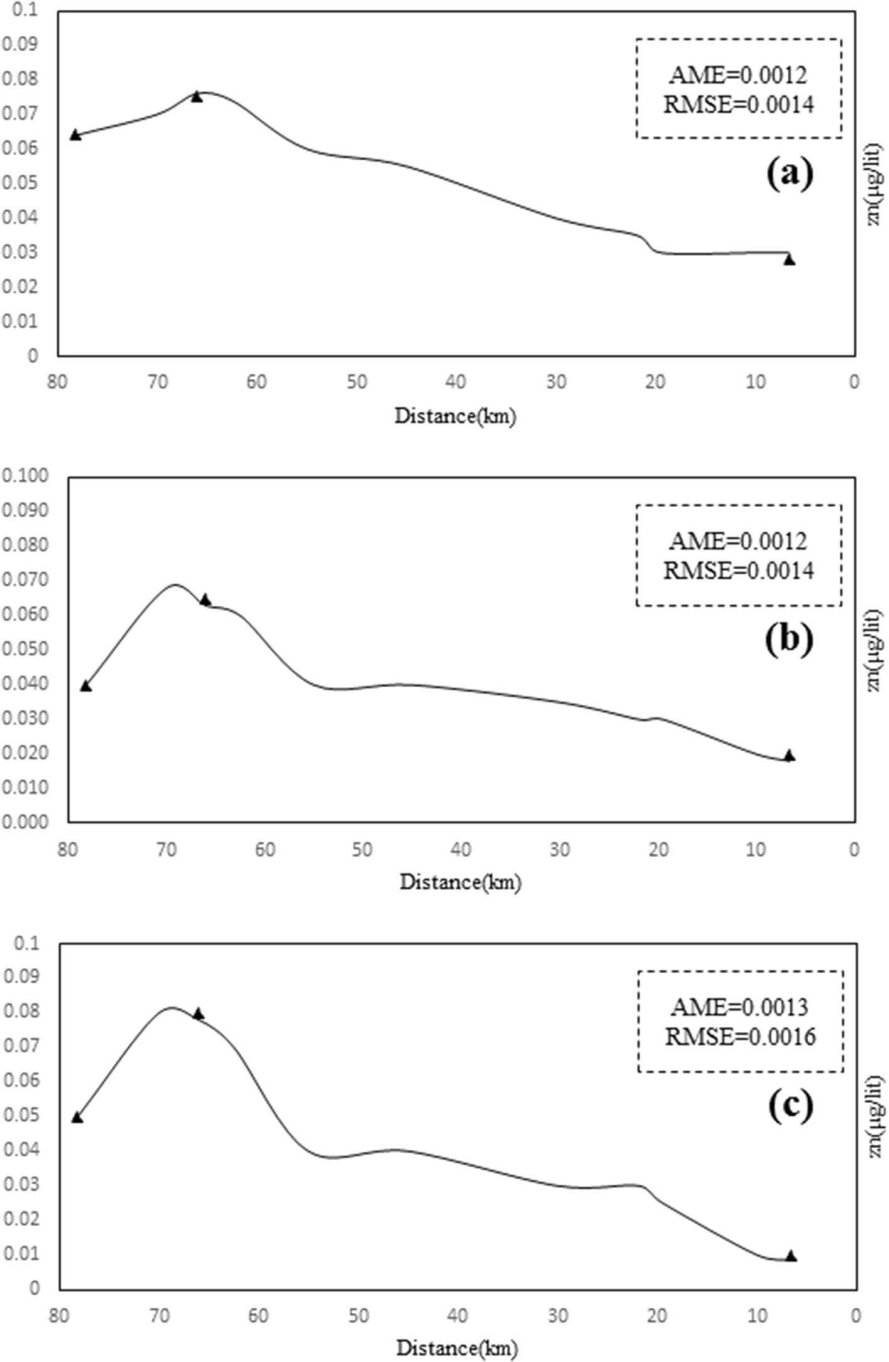
Zn Concentration on Sarouq River in (**a**) spring, (**b**) autumn, (**c**) winter in the developed Qual2kw model.

**Figure 12:**
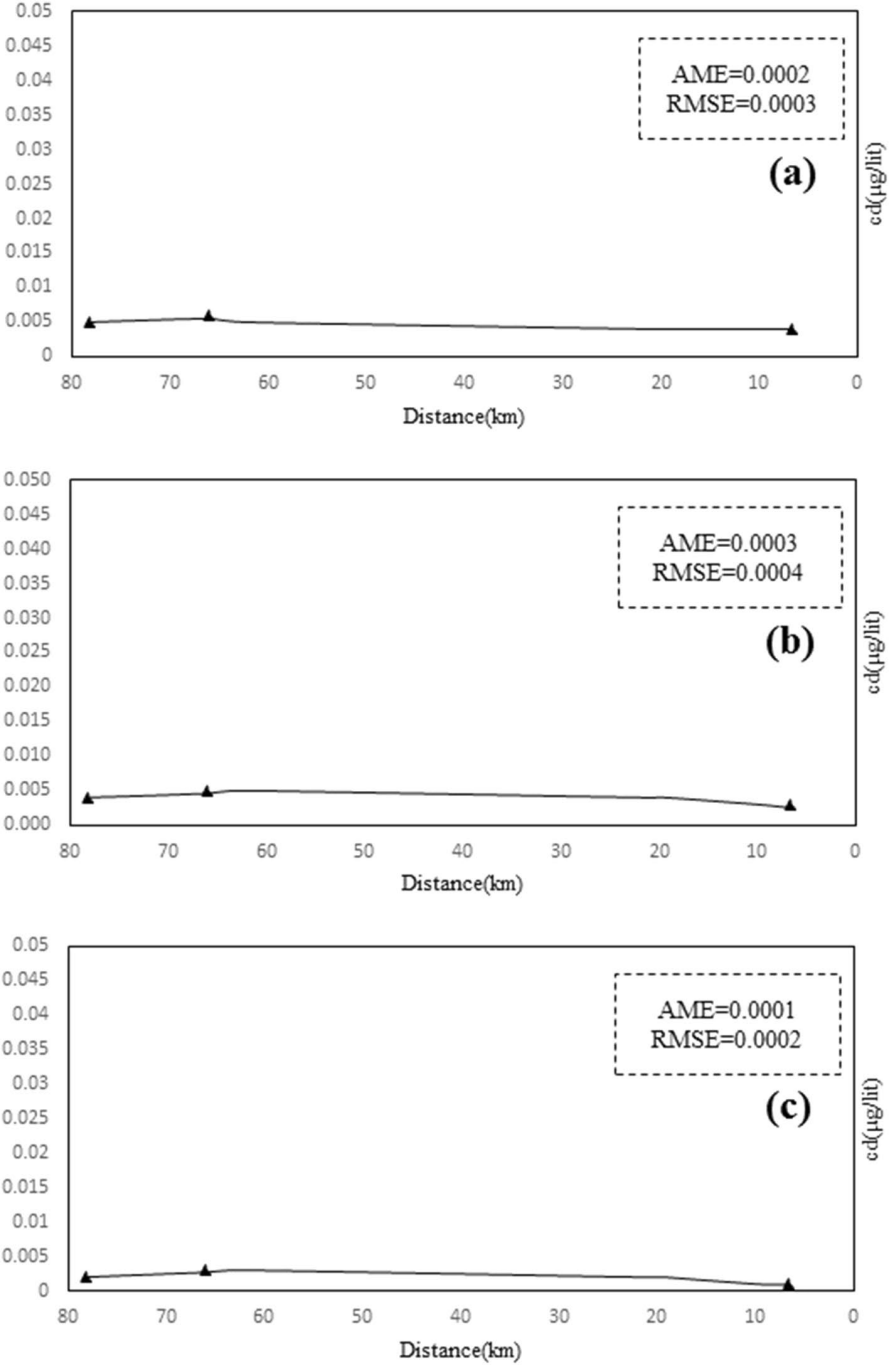
Cd Concentration on Sarouq River in (**a**) spring, (**b**) autumn, (**c**) winter in the developed Qual2kw model.

In the new heavy metal module, a linear relationship and a logarithmic relationship k were introduced in the program code part for each heavy metal, and the magnitude of the modelling error corresponds to Table 7 for the simulation of heavy metals by the reaction coefficient equations. The results presented in Table 5 show that the linear equations k are more accurate than the logarithmic equations for simulating the dissolved phase of heavy metals. Thus, using linear Eqs. 16 to 18 for the Sarouq River, the concentration values of the heavy metals of the river were derived as shown in Figs. 10, 11, 12 to simulate the concentration of the heavy metals lead, cadmium, and zinc in the dissolved phase.

**Table 7:** Calculating the amount of modeling error.

Heavy metal	Type of relationship	AME	RSME
Pb	Linear	0.31	0.46
	Logarithmic	1.8	3.7
Cd	Linear	0.24	0.42
	Logarithmic	1.2	2.3
Zn	Linear	0.56	0.75
	Logarithmic	2.8	4.8

*AME* absolute mean error.

*RMSE* root mean square error.

### Sensitivity analysis

By developing the Qual2kw model and calibrating the model, in order to investigate the effect of the quality parameters of the river on the simulation of heavy metal concentration by the developed Qual2kw model, a sensitivity analysis was performed on the model for one season of the simulation period, the results of which are in Table 8 is visible.

**Table 8:** Analysis of the sensitivity of heavy metal concentration to the quality parameters in spring.

Variable	Rate of change	Average concentration changes (%)		
		Pb	Cd	Zn
Temperature	Increase 25%	1.5	4.5	1.5
	Decrease 25%	5.2	2.4	3.25
Dissolved oxygen	Increase 25%	0.3	0.2	0.9
	Decrease 25%	0.7	0.3	1.8
Electrical conductivity	Increase 25%	3.8	10	1.3
	Decrease 25%	2.2	8.5	1.5
pH	Increase 25%	3.1	3.2	0.9
	Decrease 25%	4.7	5.1	1.3

The priority of sensitivity of heavy metals to temperature, dissolved oxygen, electrical conductivity, and pH is shown in Table 8 as follows:

For Pb: T > pH > EC > DO.

For Cd: EC > pH > T > DO.

For Zn: T > DO > pH > EC.

## Conclusions

The transport and transformation of the dissolved phase of Pb, Cd, and Zn were simulated in this study using the Qual2k model and the development of a 1D quality model in the Sarouq River. The Qual2k model was used to simulate temperature, pH, EC, and DO along with other hydrodynamic and water quality parameters. The input data for the developed heavy metal model were the output data, such as flow velocity and calibrated and validated quality parameters. The aforementioned quality parameters were assumed to have various effects on the reaction coefficient of the dissolved phases of Pb, Cd, and Zn. The coefficients of these forms were calibrated by contrasting the concentration values predicted by the developed model and the measured values of the dissolved phase of Pb, Cd, and Zn. The comparison of measured and simulated concentration values for the dissolved phase of heavy metal revealed that the linear function that included the sum of all environmental parameters had the highest accuracy. However, it’s important to consider how the other quality parameters mentioned above affect the reaction coefficient.

## Supplementary Information

Supplementary Information.

## Data Availability

The datasets used and/or analyzed during the current study available from the corresponding author on reasonable request. Correspondence and requests for materials should be addressed to M.Kh.P.
