# Supplementary material for: Simulation and modelling of heavy metals and water quality parameters in the river
Source: Sci Rep. 2023 Feb 21;13:3020. doi: 10.1038/s41598-023-29878-1 (PMC9944203; doi:10.1038/s41598-023-29878-1)
Supplement: Supplementary file 1 — Supplementary Information. [file 41598_2023_29878_MOESM1_ESM.docx]

Table S1. Model default values in genetic algorithm automatic calibration control settings

| ***Auto-calibration genetic algorithm control:*** | | |
| --- | --- | --- |
| **Random number seed** | **123456** | seed |
| **Model runs in a population (<=512)** | **50** | np |
| **Generations in the evolution** | **50** | ngen |
| **Digits to encode genotype (<=6)** | **5** | nd |
| **Crossover mode (1, 2, 3, 4, 5, 6, or 7)** | **3** | icross |
| **Crossover probability (0-1):** | **0.85** | pcross |
| **Mutation mode (1, 2, 3, 4, 5, or 6)** | **2** | imut |
| **Initial mutation rate (0-1):** | **0.005** | pmut |
| **Minimum mutation rate (0-1):** | **0.0005** | pmutmn |
| **Maximum mutation rate (0-1):** | **0.25** | pmutmx |
| **Relative fitness differential (0-1):** | **1** | fdif |
| **Reproduction plan (1, 2, or 3):** | **1** | irep |
| **Elitism (0 or 1):** | **1** | ielite |
| **Restart from previous evolution (0 or 1):** | **0** | irestart |

| ***System ID:*** |  |  |
| --- | --- | --- |
| **River name** | Sarouq |  |
| **Saved file name** | Sarouq |  |
| **Directory where the input/output files are saved** | **E:\A\model** |  |
| **Month** | **5** | **MAY** |
| **Day** | **20** |  |
| **Year** | **2008** |  |
| **Local standard time zone relative to UTC** | **-3.30** | **hours** |
| **Daylight savings time** | **Yes** |  |
| ***Simulation and output options:*** |  |  |
| **Calculation step** | **1.40625** | **minutes** |
| **Number of days** | **10** | **days** |
| **Solution method (integration)** | **Euler** |  |
| **Solution method (pH)** | **Newton-Raphson** |  |
| **Simulate hyporheic exchange and pore water quality** | **No** |  |
| **Display dynamic diel output** | **No** |  |
| **State variables for simulation** | **All** |  |
| **Simulate sediment diagenesis** | **Option 1** |  |
| **Simulate alkalinity change due to nutrient change** | **Yes** |  |
| **Write dynamic output of water quality** | **No** |  |
| **Program determined calc step** | **11.25** | **minutes** |
| **Time elapsed during last model run** | **0.18** | **minutes** |
| **Time of sunrise** | **6:17 AM** |  |
| **Time of solar noon** | **1:03 PM** |  |
| **Time of sunset** | **7:49 PM** |  |
| **Photoperiod** | **13.54** | **hours** |

Table S2. Initial settings of the model in spring

Table S3. Boundary conditions information of the Sarouq River in spring

| ***QUAL2Kw*** |  |  |  |  |  |  |
| --- | --- | --- | --- | --- | --- | --- |
| ***Stream Water Quality Model*** |  | 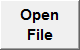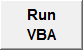   \|  \| \| --- \| |  |  | 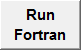   \|  \| \| --- \| |  |
| ***Sarouq*** |  |  |  |  |  |  |
| ***Headwater and Downstream Boundary Data:*** | |  |  |  |  |  |
|  |  |  |  |  |  |  |
|  |  |  |  |  |  |  |
| **Headwater Flow** | **1.710** | **m3/s** |  |  |  |  |
| **Prescribed downstream boundary?** | **Yes** |  |  |  |  |  |
| ***Headwater Water Quality*** | ***Units*** | ***12:00 AM*** | ***1:00 AM*** | ***2:00 AM*** | ***3:00 AM*** | ***4:00 AM*** |
| **Temperature** | **C** | **17.00** | **17.00** | **17.00** | **17.00** | **17.00** |
| **Conductivity** | **umhos** | **2990.00** | **2990.00** | **2990.00** | **2990.00** | **2990.00** |
| **Dissolved Oxygen** | **mg/L** | **6.90** | **6.90** | **6.90** | **6.90** | **6.90** |
| **CBODfast** | **mgO2/L** | **2.00** | **2.00** | **2.00** | **2.00** | **2.00** |
| **NO3-Nitrogen** | **ugN/L** | **0.00** | **0.00** | **0.00** | **0.00** | **0.00** |
| **Pathogen** | **cfu/100 mL** | **2400.00** | **2400.00** | **2400.00** | **2400.00** | **2400.00** |
| **Generic constituent** | **user defined** | **18.00** | **18.00** | **18.00** | **18.00** | **18.00** |
| **Alkalinity** | **mgCaCO3/L** | **100.00** | **100.00** | **100.00** | **100.00** | **100.00** |
| **pH** | **s.u.** | **7.87** | **7.87** | **7.87** | **7.87** | **7.87** |
| ***Downstream Boundary Water Quality (optional)*** | ***Units*** | ***12:00 AM*** | ***1:00 AM*** | ***2:00 AM*** | ***3:00 AM*** | ***4:00 AM*** |
| **Temperature** | **C** | **22.70** | **22.70** | **22.70** | **22.70** | **22.70** |
| **Conductivity** | **umhos** | **831.00** | **831.00** | **831.00** | **831.00** | **831.00** |
| **Dissolved Oxygen** | **mg/L** | **6.80** | **6.80** | **6.80** | **6.80** | **6.80** |
| **CBODfast** | **mgO2/L** | **2.00** | **2.00** | **2.00** | **2.00** | **2.00** |
| **NO3-Nitrogen** | **ugN/L** | **500.00** | **500.00** | **500.00** | **500.00** | **500.00** |
| **Pathogen** | **cfu/100 mL** | **130.00** | **130.00** | **130.00** | **130.00** | **130.00** |
| **Generic constituent** | **user defined** | **12.00** | **12.00** | **12.00** | **12.00** | **12.00** |
| **pH** | **s.u.** | **8.09** | **8.09** | **8.09** | **8.09** | **8.09** |

Table S4. Reach length and their geographical coordinates

| ***Reach for diel plot:*** | \|  \| \| --- \| | 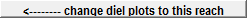 |  |  |
| --- | --- | --- | --- | --- | --- |
|  |  | ***Reach*** |  |  |
| ***Reach*** |  | ***length*** | ***Downstream*** |  |
| ***Label*** | ***Number*** | ***(km)*** | ***Latitude*** | ***Longitude*** |
|  | **0** |  | **36.37** | **47.13** |
| **1.000** | **1** | **2.92** | **36.39** | **47.11** |
| **2.000** | **2** | **2.63** | **36.41** | **47.10** |
| **3.000** | **3** | **2.39** | **36.43** | **47.10** |
| **4.000** | **4** | **4.17** | **36.45** | **47.06** |
| **5.000** | **5** | **2.31** | **36.45** | **47.04** |
| **6.000** | **6** | **2.52** | **36.44** | **47.02** |
| **7.000** | **7** | **1.49** | **36.44** | **47.01** |
| **8.000** | **8** | **1.95** | **36.44** | **46.99** |
| **9.000** | **9** | **2.05** | **36.43** | **46.97** |
| **10.000** | **10** | **3.05** | **36.42** | **46.94** |
| **11.000** | **11** | **1.45** | **36.41** | **46.93** |
| **12.000** | **12** | **2.65** | **36.42** | **46.91** |
| **13.000** | **13** | **3.92** | **36.41** | **46.88** |
| **14.000** | **14** | **4.37** | **36.42** | **46.85** |
| **15.000** | **15** | **2.73** | **36.42** | **46.84** |
| **16.000** | **16** | **2.82** | **36.41** | **46.81** |
| **17.000** | **17** | **1.98** | **36.42** | **46.81** |
| **18.000** | **18** | **2.88** | **36.42** | **46.80** |
| **19.000** | **19** | **4.09** | **36.43** | **46.77** |
| **20.000** | **20** | **2.87** | **36.42** | **46.77** |
| **21.000** | **21** | **2.16** | **36.41** | **46.76** |
| **22.000** | **22** | **2.59** | **36.41** | **46.74** |
| **23.000** | **23** | **1.89** | **36.42** | **46.73** |
| **24.000** | **24** | **2.70** | **36.41** | **46.71** |
| **25.000** | **25** | **2.98** | **36.40** | **46.69** |
| **26.000** | **26** | **2.26** | **36.40** | **46.67** |
| **27.000** | **27** | **1.70** | **36.40** | **46.66** |
| **28.000** | **28** | **4.13** | **36.40** | **46.62** |
| **29.000** | **29** | **2.49** | **36.40** | **46.60** |

Table S5.Altitude numbers of the reach and their geographic coordination

| ***Reach for diel plot:*** |  |  |  |  |  |  |  |  |  |
| --- | --- | --- | --- | --- | --- | --- | --- | --- | --- |
|  | ***Downstream*** | ***Elevation*** | | ***Downstream*** | | | | | |
| ***Reach*** | ***location*** | ***Upstream*** | ***Downstream*** | ***Latitude*** | | | ***Longitude*** | | |
| ***Label*** | ***(km)*** | ***(m)*** | ***(m)*** | ***Degrees*** | ***Minutes*** | ***Seconds*** | ***Degrees*** | ***Minutes*** | ***Seconds*** |
|  | **78.155** |  | **1820.000** | **36.00** | **22** | **26** | **47.00** | **7** | **58** |
| **1.000** | **75.235** | **1820.000** | **1800.000** | **36.00** | **23** | **39** | **47.00** | **6** | **49** |
| **2.000** | **72.600** | **1800.000** | **1780.000** | **36.00** | **24** | **45** | **47.00** | **6** | **9** |
| **3.000** | **70.208** | **1780.000** | **1760.000** | **36.00** | **25** | **49** | **47.00** | **5** | **48** |
| **4.000** | **66.037** | **1760.000** | **1740.000** | **36.00** | **27** | **10** | **47.00** | **3** | **49** |
| **5.000** | **63.729** | **1740.000** | **1720.000** | **36.00** | **26** | **55** | **47.00** | **2** | **26** |
| **6.000** | **61.210** | **1720.000** | **1700.000** | **36.00** | **26** | **37** | **47.00** | **1** | **2** |
| **7.000** | **59.722** | **1700.000** | **1680.000** | **36.00** | **26** | **8** | **47.00** | **0** | **25** |
| **8.000** | **57.768** | **1680.000** | **1660.000** | **36.00** | **26** | **11** | **46.00** | **59** | **14** |
| **9.000** | **55.716** | **1660.000** | **1650.000** | **36.00** | **25** | **45** | **46.00** | **58** | **4** |
| **10.000** | **52.670** | **1650.000** | **1640.000** | **36.00** | **24** | **57** | **46.00** | **56** | **40** |
| **11.000** | **51.219** | **1640.000** | **1630.000** | **36.00** | **24** | **42** | **46.00** | **55** | **51** |
| **12.000** | **48.569** | **1630.000** | **1620.000** | **36.00** | **24** | **55** | **46.00** | **54** | **26** |
| **13.000** | **44.647** | **1620.000** | **1610.000** | **36.00** | **24** | **27** | **46.00** | **52** | **49** |
| **14.000** | **40.278** | **1610.000** | **1605.000** | **36.00** | **24** | **55** | **46.00** | **50** | **59** |
| **15.000** | **37.546** | **1605.000** | **1600.000** | **36.00** | **25** | **5** | **46.00** | **50** | **11** |
| **16.000** | **34.723** | **1600.000** | **1595.000** | **36.00** | **24** | **41** | **46.00** | **48** | **49** |
| **17.000** | **32.742** | **1595.000** | **1585.000** | **36.00** | **25** | **15** | **46.00** | **48** | **43** |
| **18.000** | **29.859** | **1585.000** | **1575.000** | **36.00** | **25** | **27** | **46.00** | **47** | **51** |
| **19.000** | **25.771** | **1575.000** | **1565.000** | **36.00** | **25** | **42** | **46.00** | **46** | **21** |
| **20.000** | **22.905** | **1565.000** | **1555.000** | **36.00** | **24** | **56** | **46.00** | **46** | **2** |
| **21.000** | **20.744** | **1555.000** | **1535.000** | **36.00** | **24** | **36** | **46.00** | **45** | **23** |
| **22.000** | **18.152** | **1535.000** | **1500.000** | **36.00** | **24** | **48** | **46.00** | **44** | **34** |
| **23.000** | **16.259** | **1500.000** | **1490.000** | **36.00** | **25** | **14** | **46.00** | **43** | **39** |
| **24.000** | **13.556** | **1490.000** | **1480.000** | **36.00** | **24** | **40** | **46.00** | **42** | **35** |
| **25.000** | **10.573** | **1480.000** | **1470.000** | **36.00** | **24** | **12** | **46.00** | **41** | **20** |
| **26.000** | **8.316** | **1470.000** | **1460.000** | **36.00** | **23** | **50** | **46.00** | **40** | **10** |
| **27.000** | **6.618** | **1460.000** | **1450.000** | **36.00** | **24** | **7** | **46.00** | **39** | **20** |
| **28.000** | **2.489** | **1450.000** | **1440.000** | **36.00** | **24** | **0** | **46.00** | **37** | **18** |
| **29.000** | **0.000** | **1440.000** | **1430.000** | **36.00** | **24** | **1** | **46.00** | **36** | **1** |

Table S6. Hydraulic information of Sarouq river

| ***Reach for diel plot:*** |  | | | | |
| --- | --- | --- | --- | --- | --- |
|  | **Manning Formula** | | | | |
| ***Reach*** | ***Channel*** | ***Manning*** | ***Bot Width*** | ***Side*** | ***Side*** |
| ***Label*** | ***Slope*** | ***n*** | ***m*** | ***Slope*** | ***Slope*** |
|  | **0.005** | **0.0500** | **7.00** | **0.00** | **0.00** |
| **1.000** | **0.00685** | **0.0500** | **7.00** | **0.00** | **0.00** |
| **2.000** | **0.00759** | **0.0400** | **7.00** | **0.00** | **0.00** |
| **3.000** | **0.008361** | **0.0400** | **7.00** | **0.00** | **0.00** |
| **4.000** | **0.004795** | **0.0400** | **7.00** | **0.00** | **0.00** |
| **5.000** | **0.008663** | **0.0400** | **7.00** | **0.00** | **0.00** |
| **6.000** | **0.00794** | **0.0400** | **7.00** | **0.00** | **0.00** |
| **7.000** | **0.013442** | **0.0350** | **7.00** | **0.00** | **0.00** |
| **8.000** | **0.010234** | **0.0350** | **10.00** | **0.00** | **0.00** |
| **9.000** | **0.004874** | **0.0350** | **10.00** | **0.00** | **0.00** |
| **10.000** | **0.003283** | **0.0350** | **10.00** | **0.00** | **0.00** |
| **11.000** | **0.006893** | **0.0350** | **10.00** | **0.00** | **0.00** |
| **12.000** | **0.003773** | **0.0350** | **10.00** | **0.00** | **0.00** |
| **13.000** | **0.00255** | **0.0350** | **10.00** | **0.00** | **0.00** |
| **14.000** | **0.001144** | **0.0350** | **13.00** | **0.00** | **0.00** |
| **15.000** | **0.00183** | **0.0350** | **13.00** | **0.00** | **0.00** |
| **16.000** | **0.001771** | **0.0350** | **10.00** | **0.00** | **0.00** |
| **17.000** | **0.005049** | **0.0350** | **10.00** | **0.00** | **0.00** |
| **18.000** | **0.003469** | **0.0350** | **10.00** | **0.00** | **0.00** |
| **19.000** | **0.002446** | **0.0350** | **10.00** | **0.00** | **0.00** |
| **20.000** | **0.003489** | **0.0350** | **10.00** | **0.00** | **0.00** |
| **21.000** | **0.009254** | **0.0350** | **11.00** | **0.00** | **0.00** |
| **22.000** | **0.013502** | **0.0350** | **11.00** | **0.00** | **0.00** |
| **23.000** | **0.005284** | **0.0350** | **12.00** | **0.00** | **0.00** |
| **24.000** | **0.003699** | **0.0350** | **15.00** | **0.00** | **0.00** |
| **25.000** | **0.003353** | **0.0350** | **15.00** | **0.00** | **0.00** |
| **26.000** | **0.004432** | **0.0350** | **16.00** | **0.00** | **0.00** |
| **27.000** | **0.005888** | **0.0350** | **20.00** | **0.00** | **0.00** |
| **28.000** | **0.002422** | **0.0300** | **25.00** | **0.00** | **0.00** |
| **29.000** | **0.004018** | **0.0300** | **25.00** | **0.00** | **0.00** |

Table S7. Air temperature in the study area during the spring

|  | ***12:00 AM*** | ***1:00 AM*** | ***2:00 AM*** | ***3:00 AM*** | ***4:00 AM*** | ***5:00 AM*** | ***6:00 AM*** | ***7:00 AM*** |
| --- | --- | --- | --- | --- | --- | --- | --- | --- |
| ***Reach*** | ***Hourly air temperature for each reach (degrees C)*** | | | | | | | |
| ***Label*** | ***(The input values are applied as point estimates at each time. Linear interpolation is used to estimate values between the hourly inputs.)*** | | | | | | | |
| **1.00** | **19.00** | **19.00** | **19.00** | **19.00** | **19.00** | **19.00** | **19.00** | **19.00** |
| **2.00** | **20.00** | **20.00** | **20.00** | **20.00** | **20.00** | **20.00** | **20.00** | **20.00** |
| **3.00** | **21.00** | **21.00** | **21.00** | **21.00** | **21.00** | **21.00** | **21.00** | **21.00** |
| **4.00** | **23.00** | **23.00** | **23.00** | **23.00** | **23.00** | **23.00** | **23.00** | **23.00** |
| **5.00** | **24.00** | **24.00** | **24.00** | **24.00** | **24.00** | **24.00** | **24.00** | **24.00** |
| **6.00** | **24.00** | **24.00** | **24.00** | **24.00** | **24.00** | **24.00** | **24.00** | **24.00** |
| **7.00** | **24.00** | **24.00** | **24.00** | **24.00** | **24.00** | **24.00** | **24.00** | **24.00** |
| **8.00** | **24.00** | **24.00** | **24.00** | **24.00** | **24.00** | **24.00** | **24.00** | **24.00** |
| **9.00** | **24.00** | **24.00** | **24.00** | **24.00** | **24.00** | **24.00** | **24.00** | **24.00** |
| **10.00** | **24.00** | **24.00** | **24.00** | **24.00** | **24.00** | **24.00** | **24.00** | **24.00** |
| **11.00** | **24.00** | **24.00** | **24.00** | **24.00** | **24.00** | **24.00** | **24.00** | **24.00** |
| **12.00** | **24.00** | **24.00** | **24.00** | **24.00** | **24.00** | **24.00** | **24.00** | **24.00** |
| **13.00** | **24.00** | **24.00** | **24.00** | **24.00** | **24.00** | **24.00** | **24.00** | **24.00** |
| **14.00** | **24.00** | **24.00** | **24.00** | **24.00** | **24.00** | **24.00** | **24.00** | **24.00** |
| **15.00** | **24.00** | **24.00** | **24.00** | **24.00** | **24.00** | **24.00** | **24.00** | **24.00** |
| **16.00** | **24.00** | **24.00** | **24.00** | **24.00** | **24.00** | **24.00** | **24.00** | **24.00** |
| **17.00** | **24.00** | **24.00** | **24.00** | **24.00** | **24.00** | **24.00** | **24.00** | **24.00** |
| **18.00** | **24.00** | **24.00** | **24.00** | **24.00** | **24.00** | **24.00** | **24.00** | **24.00** |
| **19.00** | **24.00** | **24.00** | **24.00** | **24.00** | **24.00** | **24.00** | **24.00** | **24.00** |
| **20.00** | **24.00** | **24.00** | **24.00** | **24.00** | **24.00** | **24.00** | **24.00** | **24.00** |
| **21.00** | **24.00** | **24.00** | **24.00** | **24.00** | **24.00** | **24.00** | **24.00** | **24.00** |
| **22.00** | **24.00** | **24.00** | **24.00** | **24.00** | **24.00** | **24.00** | **24.00** | **24.00** |
| **23.00** | **24.00** | **24.00** | **24.00** | **24.00** | **24.00** | **24.00** | **24.00** | **24.00** |
| **24.00** | **24.00** | **24.00** | **24.00** | **24.00** | **24.00** | **24.00** | **24.00** | **24.00** |
| **25.00** | **24.00** | **24.00** | **24.00** | **24.00** | **24.00** | **24.00** | **24.00** | **24.00** |
| **26.00** | **24.00** | **24.00** | **24.00** | **24.00** | **24.00** | **24.00** | **24.00** | **24.00** |
| **27.00** | **24.00** | **24.00** | **24.00** | **24.00** | **24.00** | **24.00** | **24.00** | **24.00** |
| **28.00** | **25.00** | **25.00** | **25.00** | **25.00** | **25.00** | **25.00** | **25.00** | **25.00** |
| **29.00** | **25.50** | **25.50** | **25.50** | **25.50** | **25.50** | **25.50** | **25.50** | **25.50** |

Table S8. Dew point temperature in the study area in the spring

|  | ***12:00 AM*** | ***1:00 AM*** | ***2:00 AM*** | ***3:00 AM*** | ***4:00 AM*** | ***5:00 AM*** | ***6:00 AM*** | ***7:00 AM*** | ***8:00 AM*** |
| --- | --- | --- | --- | --- | --- | --- | --- | --- | --- |
| ***Reach*** | ***Hourly dew point temperature for each reach (degrees C)*** | | | | | | | | |
| ***Label*** | ***(The input values are applied as point estimates at each time. Linear interpolation is used to estimate values between the hourly inputs.)*** | | | | | | | | |
| **1.00** | **2.80** | **2.80** | **2.80** | **2.80** | **2.80** | **2.80** | **2.80** | **2.80** | **2.80** |
| **2.00** | **2.80** | **2.80** | **2.80** | **2.80** | **2.80** | **2.80** | **2.80** | **2.80** | **2.80** |
| **3.00** | **2.80** | **2.80** | **2.80** | **2.80** | **2.80** | **2.80** | **2.80** | **2.80** | **2.80** |
| **4.00** | **2.80** | **2.80** | **2.80** | **2.80** | **2.80** | **2.80** | **2.80** | **2.80** | **2.80** |
| **5.00** | **2.80** | **2.80** | **2.80** | **2.80** | **2.80** | **2.80** | **2.80** | **2.80** | **2.80** |
| **6.00** | **2.80** | **2.80** | **2.80** | **2.80** | **2.80** | **2.80** | **2.80** | **2.80** | **2.80** |
| **7.00** | **2.80** | **2.80** | **2.80** | **2.80** | **2.80** | **2.80** | **2.80** | **2.80** | **2.80** |
| **8.00** | **2.80** | **2.80** | **2.80** | **2.80** | **2.80** | **2.80** | **2.80** | **2.80** | **2.80** |
| **9.00** | **2.80** | **2.80** | **2.80** | **2.80** | **2.80** | **2.80** | **2.80** | **2.80** | **2.80** |
| **10.00** | **2.80** | **2.80** | **2.80** | **2.80** | **2.80** | **2.80** | **2.80** | **2.80** | **2.80** |
| **11.00** | **2.80** | **2.80** | **2.80** | **2.80** | **2.80** | **2.80** | **2.80** | **2.80** | **2.80** |
| **12.00** | **2.80** | **2.80** | **2.80** | **2.80** | **2.80** | **2.80** | **2.80** | **2.80** | **2.80** |
| **13.00** | **2.80** | **2.80** | **2.80** | **2.80** | **2.80** | **2.80** | **2.80** | **2.80** | **2.80** |
| **14.00** | **2.80** | **2.80** | **2.80** | **2.80** | **2.80** | **2.80** | **2.80** | **2.80** | **2.80** |
| **15.00** | **2.80** | **2.80** | **2.80** | **2.80** | **2.80** | **2.80** | **2.80** | **2.80** | **2.80** |
| **16.00** | **2.80** | **2.80** | **2.80** | **2.80** | **2.80** | **2.80** | **2.80** | **2.80** | **2.80** |
| **17.00** | **2.80** | **2.80** | **2.80** | **2.80** | **2.80** | **2.80** | **2.80** | **2.80** | **2.80** |
| **18.00** | **2.80** | **2.80** | **2.80** | **2.80** | **2.80** | **2.80** | **2.80** | **2.80** | **2.80** |
| **19.00** | **2.80** | **2.80** | **2.80** | **2.80** | **2.80** | **2.80** | **2.80** | **2.80** | **2.80** |
| **20.00** | **2.80** | **2.80** | **2.80** | **2.80** | **2.80** | **2.80** | **2.80** | **2.80** | **2.80** |
| **21.00** | **2.80** | **2.80** | **2.80** | **2.80** | **2.80** | **2.80** | **2.80** | **2.80** | **2.80** |
| **22.00** | **2.80** | **2.80** | **2.80** | **2.80** | **2.80** | **2.80** | **2.80** | **2.80** | **2.80** |
| **23.00** | **2.80** | **2.80** | **2.80** | **2.80** | **2.80** | **2.80** | **2.80** | **2.80** | **2.80** |
| **24.00** | **2.80** | **2.80** | **2.80** | **2.80** | **2.80** | **2.80** | **2.80** | **2.80** | **2.80** |
| **25.00** | **2.80** | **2.80** | **2.80** | **2.80** | **2.80** | **2.80** | **2.80** | **2.80** | **2.80** |
| **26.00** | **2.80** | **2.80** | **2.80** | **2.80** | **2.80** | **2.80** | **2.80** | **2.80** | **2.80** |
| **27.00** | **2.80** | **2.80** | **2.80** | **2.80** | **2.80** | **2.80** | **2.80** | **2.80** | **2.80** |
| **28.00** | **2.80** | **2.80** | **2.80** | **2.80** | **2.80** | **2.80** | **2.80** | **2.80** | **2.80** |
| **29.00** | **2.80** | **2.80** | **2.80** | **2.80** | **2.80** | **2.80** | **2.80** | **2.80** | **2.80** |

Table S9. Wind speed in the study area during the spring

|  | ***12:00 AM*** | ***1:00 AM*** | ***2:00 AM*** | ***3:00 AM*** | ***4:00 AM*** | ***5:00 AM*** | ***6:00 AM*** | ***7:00 AM*** | ***8:00 AM*** | ***9:00 AM*** | ***10:00 AM*** | ***11:00 AM*** |
| --- | --- | --- | --- | --- | --- | --- | --- | --- | --- | --- | --- | --- |
| ***Reach*** | ***Wind speed for each reach 7m above water surface (m/s)*** | | | | | | | | | | | |
| ***Label*** | ***(The input values are applied as point estimates at each time. Linear interpolation is used to estimate values between the hourly inputs.)*** | | | | | | | | | | | |
| **1.00** | **3.03** | **3.03** | **3.03** | **3.03** | **3.03** | **3.03** | **3.03** | **3.03** | **3.03** | **3.03** | **3.03** | **3.03** |
| **2.00** | **3.03** | **3.03** | **3.03** | **3.03** | **3.03** | **3.03** | **3.03** | **3.03** | **3.03** | **3.03** | **3.03** | **3.03** |
| **3.00** | **3.03** | **3.03** | **3.03** | **3.03** | **3.03** | **3.03** | **3.03** | **3.03** | **3.03** | **3.03** | **3.03** | **3.03** |
| **4.00** | **3.03** | **3.03** | **3.03** | **3.03** | **3.03** | **3.03** | **3.03** | **3.03** | **3.03** | **3.03** | **3.03** | **3.03** |
| **5.00** | **3.03** | **3.03** | **3.03** | **3.03** | **3.03** | **3.03** | **3.03** | **3.03** | **3.03** | **3.03** | **3.03** | **3.03** |
| **6.00** | **3.03** | **3.03** | **3.03** | **3.03** | **3.03** | **3.03** | **3.03** | **3.03** | **3.03** | **3.03** | **3.03** | **3.03** |
| **7.00** | **3.03** | **3.03** | **3.03** | **3.03** | **3.03** | **3.03** | **3.03** | **3.03** | **3.03** | **3.03** | **3.03** | **3.03** |
| **8.00** | **3.03** | **3.03** | **3.03** | **3.03** | **3.03** | **3.03** | **3.03** | **3.03** | **3.03** | **3.03** | **3.03** | **3.03** |
| **9.00** | **3.03** | **3.03** | **3.03** | **3.03** | **3.03** | **3.03** | **3.03** | **3.03** | **3.03** | **3.03** | **3.03** | **3.03** |
| **10.00** | **3.03** | **3.03** | **3.03** | **3.03** | **3.03** | **3.03** | **3.03** | **3.03** | **3.03** | **3.03** | **3.03** | **3.03** |
| **11.00** | **3.03** | **3.03** | **3.03** | **3.03** | **3.03** | **3.03** | **3.03** | **3.03** | **3.03** | **3.03** | **3.03** | **3.03** |
| **12.00** | **3.03** | **3.03** | **3.03** | **3.03** | **3.03** | **3.03** | **3.03** | **3.03** | **3.03** | **3.03** | **3.03** | **3.03** |
| **13.00** | **3.03** | **3.03** | **3.03** | **3.03** | **3.03** | **3.03** | **3.03** | **3.03** | **3.03** | **3.03** | **3.03** | **3.03** |
| **14.00** | **3.03** | **3.03** | **3.03** | **3.03** | **3.03** | **3.03** | **3.03** | **3.03** | **3.03** | **3.03** | **3.03** | **3.03** |
| **15.00** | **3.03** | **3.03** | **3.03** | **3.03** | **3.03** | **3.03** | **3.03** | **3.03** | **3.03** | **3.03** | **3.03** | **3.03** |
| **16.00** | **3.03** | **3.03** | **3.03** | **3.03** | **3.03** | **3.03** | **3.03** | **3.03** | **3.03** | **3.03** | **3.03** | **3.03** |
| **17.00** | **3.03** | **3.03** | **3.03** | **3.03** | **3.03** | **3.03** | **3.03** | **3.03** | **3.03** | **3.03** | **3.03** | **3.03** |
| **18.00** | **3.03** | **3.03** | **3.03** | **3.03** | **3.03** | **3.03** | **3.03** | **3.03** | **3.03** | **3.03** | **3.03** | **3.03** |
| **19.00** | **3.03** | **3.03** | **3.03** | **3.03** | **3.03** | **3.03** | **3.03** | **3.03** | **3.03** | **3.03** | **3.03** | **3.03** |
| **20.00** | **3.03** | **3.03** | **3.03** | **3.03** | **3.03** | **3.03** | **3.03** | **3.03** | **3.03** | **3.03** | **3.03** | **3.03** |
| **21.00** | **3.03** | **3.03** | **3.03** | **3.03** | **3.03** | **3.03** | **3.03** | **3.03** | **3.03** | **3.03** | **3.03** | **3.03** |
| **22.00** | **3.03** | **3.03** | **3.03** | **3.03** | **3.03** | **3.03** | **3.03** | **3.03** | **3.03** | **3.03** | **3.03** | **3.03** |
| **23.00** | **3.03** | **3.03** | **3.03** | **3.03** | **3.03** | **3.03** | **3.03** | **3.03** | **3.03** | **3.03** | **3.03** | **3.03** |
| **24.00** | **3.03** | **3.03** | **3.03** | **3.03** | **3.03** | **3.03** | **3.03** | **3.03** | **3.03** | **3.03** | **3.03** | **3.03** |
| **25.00** | **3.03** | **3.03** | **3.03** | **3.03** | **3.03** | **3.03** | **3.03** | **3.03** | **3.03** | **3.03** | **3.03** | **3.03** |
| **26.00** | **3.03** | **3.03** | **3.03** | **3.03** | **3.03** | **3.03** | **3.03** | **3.03** | **3.03** | **3.03** | **3.03** | **3.03** |
| **27.00** | **3.03** | **3.03** | **3.03** | **3.03** | **3.03** | **3.03** | **3.03** | **3.03** | **3.03** | **3.03** | **3.03** | **3.03** |
| **28.00** | **3.03** | **3.03** | **3.03** | **3.03** | **3.03** | **3.03** | **3.03** | **3.03** | **3.03** | **3.03** | **3.03** | **3.03** |
| **29.00** | **3.03** | **3.03** | **3.03** | **3.03** | **3.03** | **3.03** | **3.03** | **3.03** | **3.03** | **3.03** | **3.03** | **3.03** |

Table S10. Shading percentage in the study area in the spring

|  | ***Upstream*** | ***Downstream*** | ***12:00 AM*** | ***1:00 AM*** | ***2:00 AM*** | ***3:00 AM*** | ***4:00 AM*** | ***5:00 AM*** | ***6:00 AM*** | ***7:00 AM*** | ***8:00 AM*** | ***9:00 AM*** |
| --- | --- | --- | --- | --- | --- | --- | --- | --- | --- | --- | --- | --- |
| ***Reach*** | ***Distance*** | ***Distance*** | ***Integrated hourly effective shade for each reach (Percent)*** | | | | | | | | | |
| ***Number*** | ***km*** | ***km*** | ***(Percent of solar radiation that is blocked because of shade from topography and vegetation. Hourly values are applied as integrated values for each hour, e.g. the value at 12:00 AM is applied from 12:00 to 1:00 AM)*** | | | | | | | | | |
| **1** | **78.15** | **75.24** | **10.0%** | **10.0%** | **10.0%** | **10.0%** | **10.0%** | **10.0%** | **10.0%** | **10.0%** | **10.0%** | **10.0%** |
| **2** | **75.24** | **72.60** | **10.0%** | **10.0%** | **10.0%** | **10.0%** | **10.0%** | **10.0%** | **10.0%** | **10.0%** | **10.0%** | **10.0%** |
| **3** | **72.60** | **70.21** | **10.0%** | **10.0%** | **10.0%** | **10.0%** | **10.0%** | **10.0%** | **10.0%** | **10.0%** | **10.0%** | **10.0%** |
| **4** | **70.21** | **66.04** | **10.0%** | **10.0%** | **10.0%** | **10.0%** | **10.0%** | **10.0%** | **10.0%** | **10.0%** | **10.0%** | **10.0%** |
| **5** | **66.04** | **63.73** | **10.0%** | **10.0%** | **10.0%** | **10.0%** | **10.0%** | **10.0%** | **10.0%** | **10.0%** | **10.0%** | **10.0%** |
| **6** | **63.73** | **61.21** | **10.0%** | **10.0%** | **10.0%** | **10.0%** | **10.0%** | **10.0%** | **10.0%** | **10.0%** | **10.0%** | **10.0%** |
| **7** | **61.21** | **59.72** | **10.0%** | **10.0%** | **10.0%** | **10.0%** | **10.0%** | **10.0%** | **10.0%** | **10.0%** | **10.0%** | **10.0%** |
| **8** | **59.72** | **57.77** | **10.0%** | **10.0%** | **10.0%** | **10.0%** | **10.0%** | **10.0%** | **10.0%** | **10.0%** | **10.0%** | **10.0%** |
| **9** | **57.77** | **55.72** | **10.0%** | **10.0%** | **10.0%** | **10.0%** | **10.0%** | **10.0%** | **10.0%** | **10.0%** | **10.0%** | **10.0%** |
| **10** | **55.72** | **52.67** | **10.0%** | **10.0%** | **10.0%** | **10.0%** | **10.0%** | **10.0%** | **10.0%** | **10.0%** | **10.0%** | **10.0%** |
| **11** | **52.67** | **51.22** | **10.0%** | **10.0%** | **10.0%** | **10.0%** | **10.0%** | **10.0%** | **10.0%** | **10.0%** | **10.0%** | **10.0%** |
| **12** | **51.22** | **48.57** | **10.0%** | **10.0%** | **10.0%** | **10.0%** | **10.0%** | **10.0%** | **10.0%** | **10.0%** | **10.0%** | **10.0%** |
| **13** | **48.57** | **44.65** | **10.0%** | **10.0%** | **10.0%** | **10.0%** | **10.0%** | **10.0%** | **10.0%** | **10.0%** | **10.0%** | **10.0%** |
| **14** | **44.65** | **40.28** | **10.0%** | **10.0%** | **10.0%** | **10.0%** | **10.0%** | **10.0%** | **10.0%** | **10.0%** | **10.0%** | **10.0%** |
| **15** | **40.28** | **37.55** | **10.0%** | **10.0%** | **10.0%** | **10.0%** | **10.0%** | **10.0%** | **10.0%** | **10.0%** | **10.0%** | **10.0%** |
| **16** | **37.55** | **34.72** | **10.0%** | **10.0%** | **10.0%** | **10.0%** | **10.0%** | **10.0%** | **10.0%** | **10.0%** | **10.0%** | **10.0%** |
| **17** | **34.72** | **32.74** | **10.0%** | **10.0%** | **10.0%** | **10.0%** | **10.0%** | **10.0%** | **10.0%** | **10.0%** | **10.0%** | **10.0%** |
| **18** | **32.74** | **29.86** | **10.0%** | **10.0%** | **10.0%** | **10.0%** | **10.0%** | **10.0%** | **10.0%** | **10.0%** | **10.0%** | **10.0%** |
| **19** | **29.86** | **25.77** | **10.0%** | **10.0%** | **10.0%** | **10.0%** | **10.0%** | **10.0%** | **10.0%** | **10.0%** | **10.0%** | **10.0%** |
| **20** | **25.77** | **22.91** | **10.0%** | **10.0%** | **10.0%** | **10.0%** | **10.0%** | **10.0%** | **10.0%** | **10.0%** | **10.0%** | **10.0%** |
| **21** | **22.91** | **20.74** | **10.0%** | **10.0%** | **10.0%** | **10.0%** | **10.0%** | **10.0%** | **10.0%** | **10.0%** | **10.0%** | **10.0%** |
| **22** | **20.74** | **18.15** | **10.0%** | **10.0%** | **10.0%** | **10.0%** | **10.0%** | **10.0%** | **10.0%** | **10.0%** | **10.0%** | **10.0%** |
| **23** | **18.15** | **16.26** | **10.0%** | **10.0%** | **10.0%** | **10.0%** | **10.0%** | **10.0%** | **10.0%** | **10.0%** | **10.0%** | **10.0%** |
| **24** | **16.26** | **13.56** | **10.0%** | **10.0%** | **10.0%** | **10.0%** | **10.0%** | **10.0%** | **10.0%** | **10.0%** | **10.0%** | **10.0%** |
| **25** | **13.56** | **10.57** | **10.0%** | **10.0%** | **10.0%** | **10.0%** | **10.0%** | **10.0%** | **10.0%** | **10.0%** | **10.0%** | **10.0%** |
| **26** | **10.57** | **8.32** | **10.0%** | **10.0%** | **10.0%** | **10.0%** | **10.0%** | **10.0%** | **10.0%** | **10.0%** | **10.0%** | **10.0%** |
| **27** | **8.32** | **6.62** | **10.0%** | **10.0%** | **10.0%** | **10.0%** | **10.0%** | **10.0%** | **10.0%** | **10.0%** | **10.0%** | **10.0%** |
| **28** | **6.62** | **2.49** | **10.0%** | **10.0%** | **10.0%** | **10.0%** | **10.0%** | **10.0%** | **10.0%** | **10.0%** | **10.0%** | **10.0%** |
| **29** | **2.49** | **0.00** | **10.0%** | **10.0%** | **10.0%** | **10.0%** | **10.0%** | **10.0%** | **10.0%** | **10.0%** | **10.0%** | **10.0%** |

Table S11. The percentage of cloud cover in the study area in the spring

|  | ***Upstream*** | ***Downstream*** | ***12:00 AM*** | ***1:00 AM*** | ***2:00 AM*** | ***3:00 AM*** | ***4:00 AM*** | ***5:00 AM*** | ***6:00 AM*** | ***7:00 AM*** | ***8:00 AM*** |  |
| --- | --- | --- | --- | --- | --- | --- | --- | --- | --- | --- | --- | --- |
| ***Reach*** | ***Distance*** | ***Distance*** | ***Hourly cloud cover shade for each reach (Percent)*** | | | | | | | | | |
| ***Number*** | ***km*** | ***km*** | ***(Percent of sky that is covered by clouds. The input values are applied as point estimates at each time. Linear interpolation is used to estimate values between the hourly inputs.)*** | | | | | | | | | |
| **1** | **78.15** | **75.24** | **30.0%** | **30.0%** | **30.0%** | **30.0%** | **30.0%** | **30.0%** | **30.0%** | **30.0%** | **30.0%** |  |
| **2** | **75.24** | **72.60** | **30.0%** | **30.0%** | **30.0%** | **30.0%** | **30.0%** | **30.0%** | **30.0%** | **30.0%** | **30.0%** |  |
| **3** | **72.60** | **70.21** | **30.0%** | **30.0%** | **30.0%** | **30.0%** | **30.0%** | **30.0%** | **30.0%** | **30.0%** | **30.0%** |  |
| **4** | **70.21** | **66.04** | **20.0%** | **20.0%** | **20.0%** | **20.0%** | **20.0%** | **20.0%** | **20.0%** | **20.0%** | **20.0%** |  |
| **5** | **66.04** | **63.73** | **20.0%** | **20.0%** | **20.0%** | **20.0%** | **20.0%** | **20.0%** | **20.0%** | **20.0%** | **20.0%** |  |
| **6** | **63.73** | **61.21** | **10.0%** | **10.0%** | **10.0%** | **10.0%** | **10.0%** | **10.0%** | **10.0%** | **10.0%** | **10.0%** |  |
| **7** | **61.21** | **59.72** | **10.0%** | **10.0%** | **10.0%** | **10.0%** | **10.0%** | **10.0%** | **10.0%** | **10.0%** | **10.0%** |  |
| **8** | **59.72** | **57.77** | **10.0%** | **10.0%** | **10.0%** | **10.0%** | **10.0%** | **10.0%** | **10.0%** | **10.0%** | **10.0%** |  |
| **9** | **57.77** | **55.72** | **10.0%** | **10.0%** | **10.0%** | **10.0%** | **10.0%** | **10.0%** | **10.0%** | **10.0%** | **10.0%** |  |
| **10** | **55.72** | **52.67** | **10.0%** | **10.0%** | **10.0%** | **10.0%** | **10.0%** | **10.0%** | **10.0%** | **10.0%** | **10.0%** |  |
| **11** | **52.67** | **51.22** | **10.0%** | **10.0%** | **10.0%** | **10.0%** | **10.0%** | **10.0%** | **10.0%** | **10.0%** | **10.0%** |  |
| **12** | **51.22** | **48.57** | **10.0%** | **10.0%** | **10.0%** | **10.0%** | **10.0%** | **10.0%** | **10.0%** | **10.0%** | **10.0%** |  |
| **13** | **48.57** | **44.65** | **10.0%** | **10.0%** | **10.0%** | **10.0%** | **10.0%** | **10.0%** | **10.0%** | **10.0%** | **10.0%** |  |
| **14** | **44.65** | **40.28** | **10.0%** | **10.0%** | **10.0%** | **10.0%** | **10.0%** | **10.0%** | **10.0%** | **10.0%** | **10.0%** |  |
| **15** | **40.28** | **37.55** | **10.0%** | **10.0%** | **10.0%** | **10.0%** | **10.0%** | **10.0%** | **10.0%** | **10.0%** | **10.0%** |  |
| **16** | **37.55** | **34.72** | **10.0%** | **10.0%** | **10.0%** | **10.0%** | **10.0%** | **10.0%** | **10.0%** | **10.0%** | **10.0%** |  |
| **17** | **34.72** | **32.74** | **10.0%** | **10.0%** | **10.0%** | **10.0%** | **10.0%** | **10.0%** | **10.0%** | **10.0%** | **10.0%** |  |
| **18** | **32.74** | **29.86** | **10.0%** | **10.0%** | **10.0%** | **10.0%** | **10.0%** | **10.0%** | **10.0%** | **10.0%** | **10.0%** |  |
| **19** | **29.86** | **25.77** | **10.0%** | **10.0%** | **10.0%** | **10.0%** | **10.0%** | **10.0%** | **10.0%** | **10.0%** | **10.0%** |  |
| **20** | **25.77** | **22.91** | **10.0%** | **10.0%** | **10.0%** | **10.0%** | **10.0%** | **10.0%** | **10.0%** | **10.0%** | **10.0%** |  |
| **21** | **22.91** | **20.74** | **10.0%** | **10.0%** | **10.0%** | **10.0%** | **10.0%** | **10.0%** | **10.0%** | **10.0%** | **10.0%** |  |
| **22** | **20.74** | **18.15** | **10.0%** | **10.0%** | **10.0%** | **10.0%** | **10.0%** | **10.0%** | **10.0%** | **10.0%** | **10.0%** |  |
| **23** | **18.15** | **16.26** | **10.0%** | **10.0%** | **10.0%** | **10.0%** | **10.0%** | **10.0%** | **10.0%** | **10.0%** | **10.0%** |  |
| **24** | **16.26** | **13.56** | **10.0%** | **10.0%** | **10.0%** | **10.0%** | **10.0%** | **10.0%** | **10.0%** | **10.0%** | **10.0%** |  |
| **25** | **13.56** | **10.57** | **10.0%** | **10.0%** | **10.0%** | **10.0%** | **10.0%** | **10.0%** | **10.0%** | **10.0%** | **10.0%** |  |
| **26** | **10.57** | **8.32** | **10.0%** | **10.0%** | **10.0%** | **10.0%** | **10.0%** | **10.0%** | **10.0%** | **10.0%** | **10.0%** |  |
| **27** | **8.32** | **6.62** | **10.0%** | **10.0%** | **10.0%** | **10.0%** | **10.0%** | **10.0%** | **10.0%** | **10.0%** | **10.0%** |  |
| **28** | **6.62** | **2.49** | **10.0%** | **10.0%** | **10.0%** | **10.0%** | **10.0%** | **10.0%** | **10.0%** | **10.0%** | **10.0%** |  |
| **29** | **2.49** | **0.00** | **10.0%** | **10.0%** | **10.0%** | **10.0%** | **10.0%** | **10.0%** | **10.0%** | **10.0%** | **10.0%** |  |
